# On the importance of the hip abductors during a clinical one legged balance test: A theoretical study

**DOI:** 10.1371/journal.pone.0242454

**Published:** 2020-11-13

**Authors:** Payam Mirshams Shahshahani, James A. Ashton-Miller

**Affiliations:** 1 Department of Mechanical Engineering, University of Michigan, Ann Arbor, Michigan, United States of America; 2 Department of Biomedical Engineering, University of Michigan, Ann Arbor, Michigan, United States of America; 3 School of Kinesiology, University of Michigan, Ann Arbor, Michigan, United States of America; 4 Institute of Gerontology, University of Michigan, Ann Arbor, Michigan, United States of America; Toronto Rehabilitation Institute - UHN, CANADA

## Abstract

**Background:**

The ability to balance on one foot for a certain time is a widely used clinical test to assess the effects of age and diseases like peripheral neuropathy on balance. While state-space methods have been used to explore the mechanical demands and achievable accelerations for balancing on two feet in the sagittal plane, less is known about the requirements for sustaining one legged balance (OLB) in the frontal plane.

**Research question:**

While most studies have focused on ankle function in OLB, can age and/or disease-related decreases in maximum hip abduction strength also affect OLB ability?

**Methods:**

A two-link frontal plane state space model was used to define and explore the ‘feasible balance region’ which helps reveal the requirements for maintaining and restoring OLB, given the adverse effects of age and peripheral neuropathy on maximum hip and ankle strengths.

**Results:**

Maintaining quasistatic OLB required 50%-106% of the maximum hip abduction strength in young and older adults, and older patients with peripheral neuropathy. Effectiveness of a ‘hip strategy’ in recovering OLB was heavily dependent on the maximum hip abduction strength, and for healthy older women was as important as ankle strength. Natural reductions of strength due to healthy aging did not show a meaningful reduction in meeting the strength requirement of clinical OLB. However deficits in hip strength typical of patients with peripheral neuropathy did adversely affect both quasistatic OLB and recoverable OLB states.

**Significance:**

The importance of hip muscle strength has been underappreciated in the clinical OLB test. This is partly because the passive tissues of the hip joint can mask moderate deficits in hip abduction strength until it is needed for recovering OLB. Adding a follow up OLB test with a slightly raised pelvis would be a simple way to check for adequate hip abductor muscle strength.

## 1. Introduction

One-legged balance (OLB) is a familiar activity of daily living, as for example when pulling on a pant leg or sock. Because it is familiar, the ability to balance on one leg is commonly used to test balance in the clinic, especially since the timed ability to stand on one leg declines with age, as well as with diseases that affect neuromuscular coordination and sensorimotor function [1, 2]. During such a test, a clinician asks the patient to lift one foot off the ground without touching it against the stance leg and then measures how long balance can be maintained, recording the outcome as the unipedal stance time (UST). Depending upon the protocol being followed, the performance is scored as perfect (and halted) if the patient reaches the stated goal, which often ranges from 30 s to two minutes [1, 2]. On the other hand, thresholds for minimum UST performance of five seconds [3] or 30 s [4] have been proposed to identify patients at higher risk for injurious falls. The clinician also notes severe weakness in the ipsilateral hip abductor muscles during the OLB trial if the pelvis significantly drops on the contralateral side (positive Trendelenburg sign), or the trunk is visibly shifted toward the ipsilateral hip to reduce the hip abduction moment demand (negative Trendelenburg sign) [5]. In some studies a weak but significant correlation between UST and maximum hip abduction strength has been noted [6] and this relationship strengthens with age and peripheral neuropathy (PN) [7]. These results suggest a threshold may exist for the hip abduction strength in order for a patient to perform well in the OLB test. What that threshold may be is unknown; moreover, that knowledge gap is an impediment to setting rehabilitation goals for that patient.

Biomechanical studies of OLB have traditionally focused on the stance foot and the ankle joint [8–11]. A torque developed at the ankle controls the position of the resultant vertical reaction force under the stance foot, namely the center of pressure (COP) [8, 10, 12]. We can call this the ‘*ankle strategy*’ due to its similarity to its role in the well-studied eponymous strategy in bipedal standing [13]. Physically, the COP cannot be moved outside the area under the stance foot, the base of support (BOS). If the ankle muscles are strong enough, the subject can bring the COP close to the margins of the BOS. But any further exertion and the foot will tilt onto its edge thereby saturating the effective ankle moment. On the other hand if the ankle muscles are weak, they cannot move the COP as far. The “***Functional Base of Support***” (**FBOS**) or other variations of the term have been used to demonstrate this relationship [14, 15].

In this and the next paragraph let us first consider the strength requirements for maintaining quasistatic balance. For purely static balance, to satisfy mechanical equilibrium conditions [16], the vertical projection of the center of mass (COM) onto the floor has to coincide with the COP. Any offset between the two will accelerate the COM away from the COP [17, 18]. Since OLB is an unstable equilibrium and the ankle strategy is the only way to control OLB while staying quasistatic [12], maintaining a large enough FBOS width can then be considered an ankle strength requirement for quasistatic OLB [9, 14]. This is especially true given that successful OLB trials are, for the most part, quasistatic [11]. However, even when quasistatic, there should be enough strength about all the joints to maintain a given OLB posture. For example, during OLB, the COM of the body without the stance leg stays medial to the ipsilateral hip, thereby creating a large hip abduction moment demand.

The hip abduction moment required for quasistatic OLB received attention in the mid-twentieth century when it was important for calculating the contact loads on the first prosthetic hips. Notably, Inman [19] theoretically calculated the hip abduction moment to be equal to the weight of the person times half of the distance between the femoral head centers; the pelvis was assumed to be kept level. Then in a clever experiment, Inman [19] calibrated the hip abductor muscles activity, using both needle and surface EMG, as a function of measured hip abduction moment exertions. The participants then stood on the same leg, while EMG activity was being recorded to estimate the hip abduction moment. Surprisingly, Inman’s estimates of the hip abduction moment based on the measured EMG activity were almost half of his theoretical calculations. Furthermore, decreasing the pelvic inclination angle *decreased* the EMG activity in spite of the fact that doing so actually increased the hip abduction moment demand. (In the present paper decreasing pelvic inclination angle is defined as lowering the contralateral hip below a horizontal line through the ipsilateral hip, and vice versa.) Inman [19] concluded that this discrepancy was due to tension in the iliotibial band. About twenty years later, Charnley and McLeish [20] confirmed Inman’s theoretical calculations for the hip abduction moment for a level pelvis. Charnley and McLeish [20] then went on, in an experiment with three subjects, to show that the hip abduction moment demand greatly depended on the pelvic inclination angle and lateral bending of the torso. However, they refuted Inman’s theory of iliotibial tension on two grounds: first, subjects did not achieve the end of their hip adduction range of motion while standing on one leg which seemed necessary to engage the passive iliotibial tissues and, second, they dismissed Inman’s EMG evidence as faulty measurements due to old amplifier technology as well as a failure to account for changes in muscle length. A 2014 study with EMG recordings from gluteal muscles [21], however, corroborated the same trend of increased muscle activity with increasing the pelvic inclination angle, and vice versa, that Inman had observed. So, it is clear that both the hip abduction moment demand of OLB, as well as the relative contribution of passive tissues and muscles in meeting the demand, change as a function of pelvic inclination angle. However, to our knowledge, the relative contributions of the hip abductor muscles and passive abductor tissues toward meeting the hip abduction moment demand in OLB is currently unknown. This gap has confounded the ability to estimate the hip abduction strength requirements of OLB and has also made interpretation of the clinical OLB test result more difficult.

Now let us consider what happens when maintaining balance quasistatically is no longer possible. What dynamic strategies could be used to recover balance? A rapid change in hip abduction moment is a part of a more dynamic set of strategies for controlling the COM during OLB. For example, a change in angular momentum of the body, developed by creating rapid movements of the torso and non-stance limbs, can create shear forces under the stance foot that affect the COM movements via the ‘*shear force strategy*’ [12]. Otten [22] showed that the stance limb hip is the most effective joint in creating larger instantaneous shear forces under the stance foot. In the same way one can analyze control strategies for bipedal standing in the sagittal plane [13], we can call this the ‘hip strategy’ if we assume the rest of the body is kept in the same relative position for the duration of the OLB trial. While such a dynamic strategy is only used by subjects as a “plan B”, after the ankle strategy [11], it should still be considered an important part of OLB test when considering its potential for helping to avoid lateral falls [23].

Sufficient hip abduction strength is important for both efficacy of the hip strategy for controlling balance as well as maintaining quasistatic OLB. In what follows we use a double inverted pendulum mass-link model to investigate the effect of hip abduction strength on both modalities. To study age and disease effects, we applied scaled ankle inversion / eversion and hip abduction / adduction strength data from three different population groups to our model, namely healthy younger adults, healthy older adults, and older patients with PN, the latter were chosen because they are a large group of patients with known balance problems [24].

We will first explore how hip abduction strength affects quasistatic OLB by introducing the ‘***Feasible Balance Region***’ (**FBR**) as a tool to capture the different states in which one meets the strength requirements of quasistatic OLB. FBR is essentially an extension of the FBOS that includes the hip abduction strength requirement as well. The state variable approach was introduced by Kuo and Zajac [25] to determine ‘feasible accelerations sets’ in the ankle and hip angle state-space in the sagittal plane during bipedal standing as a function of individual muscle capacities. Similarly, Pai and Patton [26] used a ‘feasible stability region’ to examine the effect of maximum ankle torque, foot-floor friction, and BOS length on the ability to attain bipedal quasistatic balance. Here we build on these earlier state-space approaches to show the effect of considering a reasonable lower bound for the passive tissue hip abduction moment on maintaining quasistatic OLB, by both including and excluding it in order to arrive at the net and active FBR, respectively. Finally, for two groups of subjects with similar net FBR, we show that the active FBR is a better representation of the effectiveness of the hip strategy for avoiding an ipsilateral fall from OLB. We will conclude by discussing how our findings can be used to better interpret the results of the clinical OLB test.

## 2. Methods

### 2.1. Modeling of OLB in the frontal plane with a double inverted pendulum

**Fig 1:**
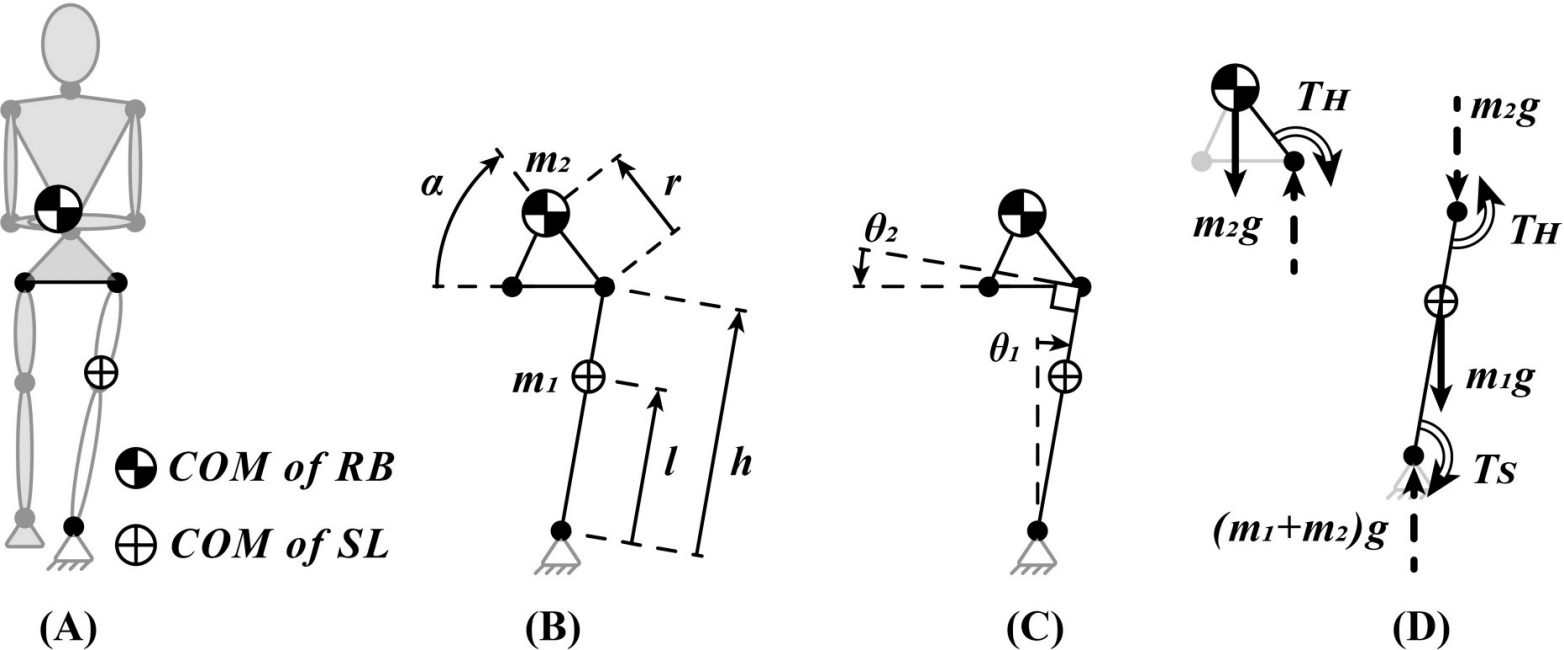
Details of the double inverted pendulum model used to represent the task of balancing on one leg in the frontal plane over a stationary stance foot. **(A)** One link represents the stance leg (SL) and the other the rest of the body (RB). The trunk is assumed to be straight without any lateral bending, and the contralateral leg is kept at a neutral abduction angle. **(B)** Parameters of the model are calculated from published mass-link information of a mid-size male aviator (m_1_ = 14 Kg, m_2_ = 67 Kg, l = 57 cm, h = 88 cm, r = 19 cm, α = 56°) [29]. **(C)** Two independent states *(θ*_*1*_, *θ*_*2*_*)* are required to describe balancing quasistatically on one leg. **(D)** Free Body Diagram for the equilibrium of the quasistatic double inverted pendulum.

Details of the double inverted pendulum model of balancing on one leg and some of the assumptions of the model can be seen in Fig 1. Maximum isometric strengths for ankle inversion / eversion, and hip abduction / adduction in neutral position were found from the literature for men and women in three different groups to represent the effect of age and neuropathy on OLB: healthy younger adults (20–30 years), healthy older adults (65+ years), and older patients with PN [7, 27, 28]. In order to apply the isometric strengths to our model, they were first normalized by the reported mean mass and height of the subjects in each group, and scaled back to the mass and height of the model (please see S1 Text). Maximum ankle inversion / eversion strengths were considered constant, and passive tissue contribution as negligible within the ankle angles common during OLB.

### 2.2. Available hip abduction moment within the range of motion

The net hip abduction moment consist of both active and passive components. The active hip abduction moment is generated by the abductor muscles; the passive moment is generated by non-muscular tissues surrounding the hip such as ligaments and fasciae. The net effect of force-length changes in the muscles of the hip and their corresponding moment arms within the range of motion was accounted for by using Neuman’s reported changes in hip abduction / adduction isometric strength as a function of hip abduction angle [30].

Our review of the literature revealed no studies for which the passive hip abduction moment had been modeled as a function of the hip abduction angle. However, an exponential relationship has been suggested for the passive hip flexion / extension moment as a function of hip flexion angle [31, 32]. Since the range of hip abduction angles encountered during clinical OLB is nowhere near the hip abduction end of range of motion [33], we assumed the passive hip adduction moment during OLB was negligible. On the other hand, whether passive hip abduction moment during OLB is negligible is a subject of debate [20]. We therefore considered the passive hip abduction moment to follow an exponential trend as a function of hip abduction angle (*θ*_*2*_):

$T_{passive}=B_{1}e^{k_{1}\theta_{2}}$, where *θ*_*2*_ is the hip abduction angle (degrees) and T_passive_ in N.m. We fit the exponential passive tissue moment for the hip abduction based on two studies. First, in a study of passive hip range of motion, Nussbaumer *et al*. [34] reported a mean abduction moment of 1.2 ± 0.36 N.m/º at the end of passive adduction range of motion (~22º adduction) for their 15 healthy subjects. Second, van Arkle *et al*. [35] reported an abduction moment of 5 N.m at about 10º of adduction in a cadaver study with all tissue being removed except for the capsular ligaments of the hip. B_1_ and k_1_ were found to be 1.276 and -0.137 respectively by fitting the curve to passive abduction moments in the aforementioned studies. Since the passive tissue moment at lower adduction angle was based on a cadaver study with only capsular ligaments intact, it is safe to assume this formula as a lower limit to the passive hip abduction moment.

### 2.3. Quasistatic OLB and feasible balance region

Two variables *(θ*_*1*_, *θ*_*2*_*)* are sufficient to describe all the states in our model for quasistatic OLB (Fig 1C). Ankle eversion moment (T_S_^+^) and the hip abduction moment (T_H_^+^) required for maintaining a quasistatic OLB state can be derived from the equilibrium equations (please see S2 Text). For a description of parameters in Eqs (1) and (2) please refer to Fig 1B. Eq (3) limits the range of pelvic inclination angles between contralateral hip sag and raise encountered in the clinic [19].

$$T_{S}(\theta_{1},\theta_{2})=-(m_{1}l+m_{2}h)g\;\sin\;\theta_{1}+m_{2}gr\;\cos\left(\alpha +\theta_{1}+\theta_{2}\right)$$ (1)

$${T_{H}(\theta_{1},\theta_{2})=m}_{2}gr\;\cos\left(\alpha +\theta_{1}+\theta_{2}\right)$$ (2)

$$-20{}^{\circ}\leq \theta_{1}+\theta_{2}\leq 20{}^{\circ}$$ (3)

The combination of states within the physiological range of motion where the strength requirements of maintaining quasistatic OLB is possible depends on the maximum available moment at the ankle and stance hip. We assumed the reported maximum ankle strengths correspond to ankle moments that would bring the center of pressure to the lateral and medial edges of the functional base of support. Since our focus is on the hip abduction moment demand in this study, this assumption will have negligible effects on the results. Combining these constraints results in an area in the *(θ*_*1*_, *θ*_*2*_*)* plane where quasistatic one-legged balance can be maintained. We refer to this area as the Feasible Balance Region (FBR). FBR calculated with only active hip abduction strength is called the *active FBR*. FBR calculated with both active and passive hip abduction moments will be referred to as the *net FBR*.

### 2.4. Effectiveness of hip strategy for recovering OLB

Lateral falls onto the stance hip are more likely to cause injury compared to medial falls where a person can simply place their contralateral foot down to break the momentum [23]. Initial quasistatic states where the COM stays lateral to the stance ankle would lead to a lateral fall if a corrective strategy was not initiated. A simple criterion for a successful recovery from a lateral fall is to find a strategy that causes the double inverted pendulum model to fall medially instead. Using MATLAB® simulations of the equations of motion for the double inverted pendulum model of OLB (*ode45 solver*), we found all the initial quasistatic OLB states where recovering OLB was possible (please see S2 Text for the derivation of the equations of motion using Newtonian Mechanics). We implemented a simple control strategy in which to avoid a lateral fall, maximum ankle inversion and maximum hip abduction moments are activated from their initial values. This strategy can be derived both specifically for the double inverted pendulum (section 4 in S2 Text) and generally based on the induced change in angular momentum of the body for a multi-link model, for example see Hof (2007) [12]. Range of motion constraint was implemented by adding a large negative damping term in the last two degrees of the range of motion. We assumed zero initial ankle moment (T_S0_ = 0), and the smaller of two values for the initial hip abduction moment (T_H0_); the one calculated from Eq (2) and the maximum net available hip abduction moment at the initial state. A limited rate of torque development for both ankle and hip moments was enforced based the work of Thelen *et al*. [36]; briefly, they showed for ankle plantarflexor moment that regardless of age or sex, subjects took on average 90 ms to increase the moment from rest to half of their maximum strength. For more details on the assumptions of these simulations, please refer to S3 Text.

## 3. Results

### 3.1. Calculated hip abduction moment demand for OLB

The hip abduction moment demand for standing on one leg was calculated assuming the pelvis was kept horizontal *(θ*_*1*_*+ θ*_*2*_ *= 0)*. For men in the healthy younger, healthy older, and older with peripheral neuropathy groups, the hip abduction moment demand for OLB was calculated to be 50%, 66%, and 106% of their maximum voluntary isometric hip abduction strength respectively. For women in the aforementioned groups, the calculated values for the hip abduction moment demand for OLB were 57%, 82%, and 95% of their maximum voluntary isometric hip abduction strength, respectively.

### 3.2. Active feasible balance region (FBR) for quasistatic OLB

**Fig 2:**
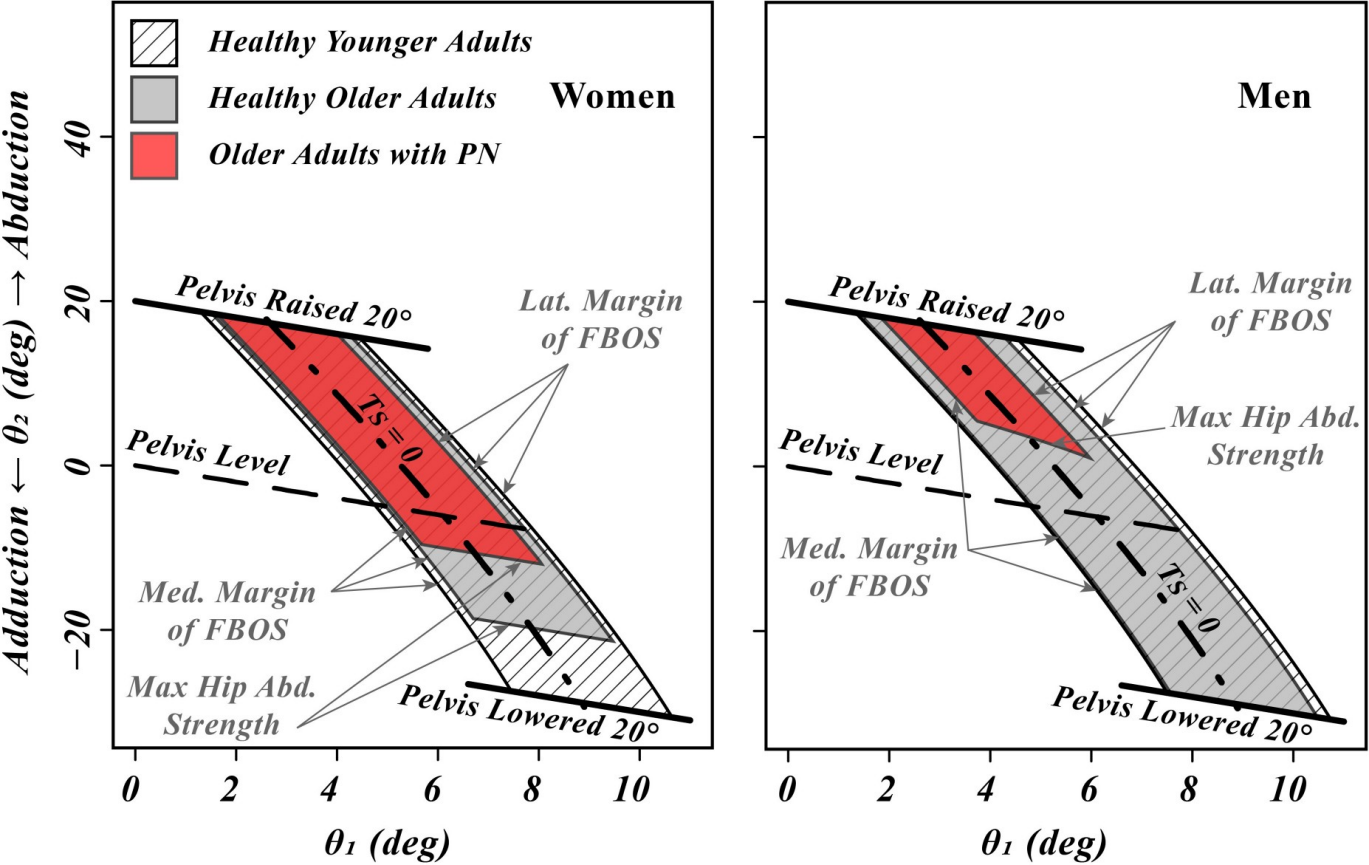
A graphical comparison of the effect of ankle and hip strength on the mean calculated quasistatic active feasible balance region (FBR) for OLB in women (left) and men (right) of different ages and health status. A point on each state-space plot represents a quasistatic state for the OLB double inverted pendulum model with a fixed foot link, where *θ*_*1*_ is the angle that the stance leg makes with the vertical line and *θ*_*2*_ is the stance leg’s hip abduction angle (please see Fig 1C). Because of limited ankle and hip strengths and ranges of motion, quasistatic OLB can only be maintained for the states inside the solid lines defining the FBR for each sex and group. The dashed-dot line in the middle of each FBR is the locus of states for which the COM lies in the same vertical plane as the ankle so that no ankle moment is required (T_S_ = 0). The top boundary of the FBR for all groups is the locus of points for which the pelvic inclination angle is raised a nominal 20º. For healthy young men and women, and healthy older men, the lower boundary of the FBR is the locus of points for which the pelvic inclination angle is decreased a nominal 20º. However, for healthy older women and older patients with peripheral neuropathy it is the maximum active hip abduction strength that constrains the lower end of the FBR. The left and right boundaries of the FBR are always determined by the medial and lateral margins of the functional base of support, respectively.

Fig 2 shows the predicted active FBR for each of the populations. The sensitivity of the calculated hip abduction moment demand and the active FBR to lateral bending of the trunk and movements of the contralateral leg are given in S4 Text.

### 3.3. Effect of including hip abduction passive tissue moment on the FBR

**Fig 3:**
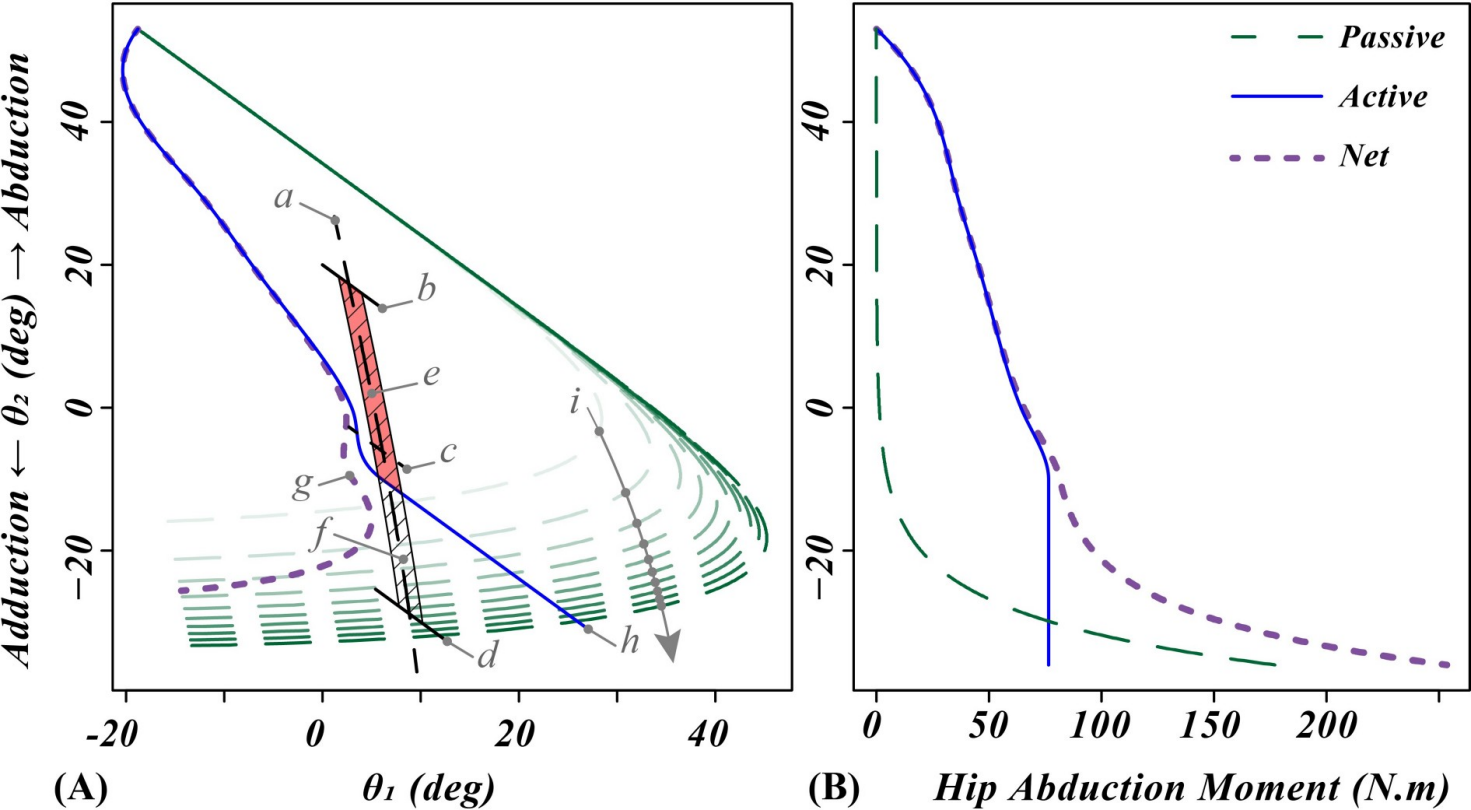
Example of calculated passive tissue contribution to hip abduction moment during OLB in older women with peripheral neuropathy. **(A)** Calculated effect of passive tissue contribution to the net hip abduction moment on the FBR. Each line in the plot tagged with a small letter in gray shows a locus of quasistatic OLB states where a condition is satisfied: a: zero ankle moment (T_S_ = 0); b: pelvic inclination angle increased 20º; c: pelvic inclination angle held level with horizon; d: pelvic inclination angle decreased to -20º; e: FBR derived with only active hip abduction strength (red region); f: FBR derived with both active and passive hip abduction moments (region with diagonal lines); g: required hip abduction moment for maintaining the quasistatic state is equal to the net available hip abduction moment; h: required hip abduction moment for maintaining the quasistatic state is equal to the maximum active hip abduction moment strength; i: each green dashed line shows the states where the hip abduction moment from the passive tissues is equal to a percentage of the hip abduction moment load of OLB ranging from 10% to 100% in 10% increments which increase in the direction of the arrow. **(B)** Available hip abduction moment as a function of hip abduction angle.

**Fig 4:**
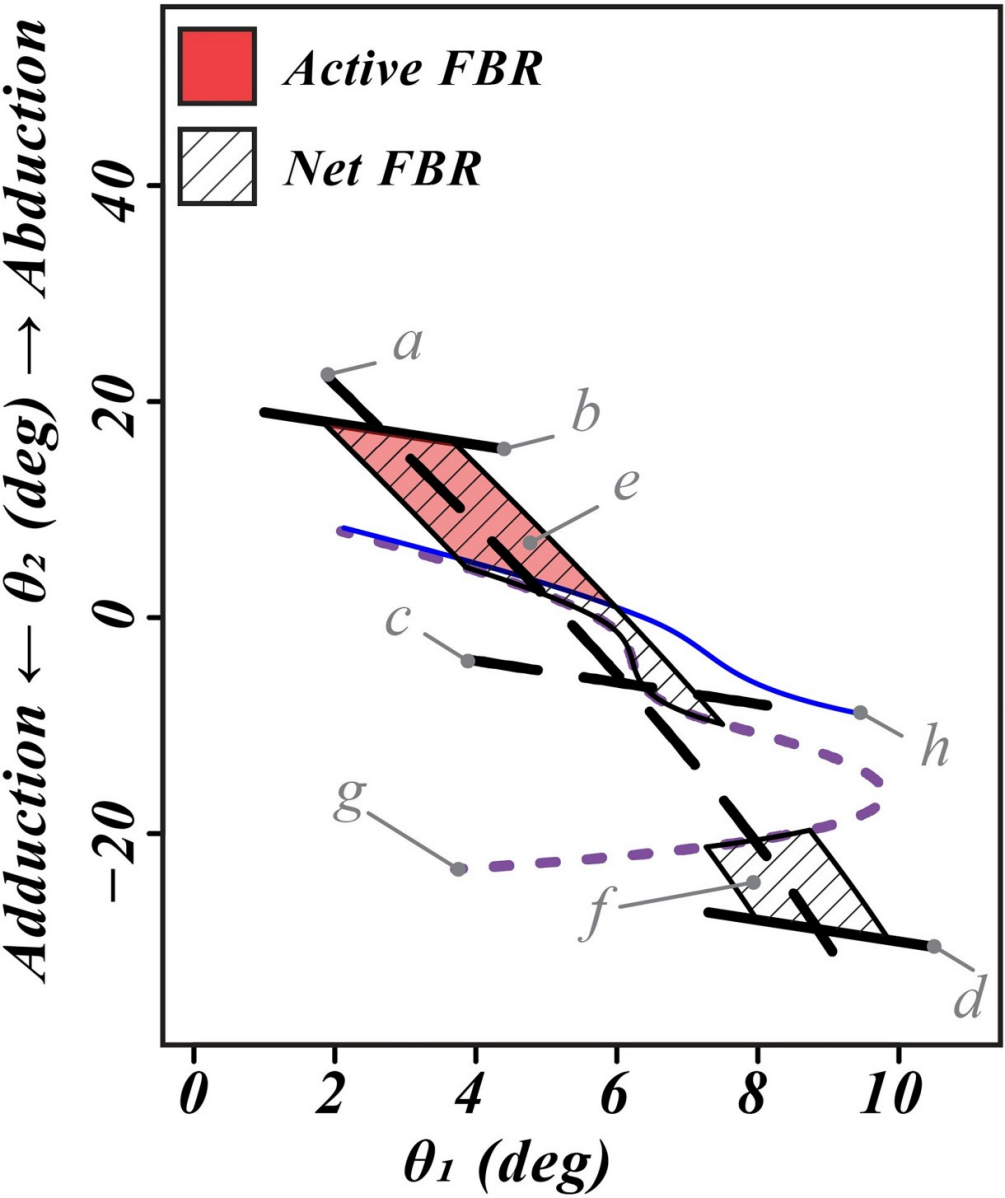
Example of passive tissue contribution to hip abduction moment during OLB in older men with PN. Calculated effect of significantly decreased active hip abduction strength on the FBR for older men with peripheral neuropathy. The locus of points tagged by grey letters a-g and the choice of colors are the same as described in Fig 3A.

If we include the modeled contribution of passive tissues to the net available hip abduction moment, the net FBR for all groups, except for the older men with PN, would extend between the lines for pelvic inclination angles decreased and increased by 20º on Fig 2. For example, Fig 3 shows the increased engagement of the passive tissues in bearing more of the hip abduction moment demand with increasing hip adduction (smaller *θ*_*2*_ values) in older women with PN. Fig 4 shows that for the corresponding population of older men with PN, the passive tissue contribution to the net available hip abduction moment was not enough to compensate for the weakness of the hip abductor muscles, leading to the net FBR actually being divided into two parts.

### 3.4. Effectiveness of the hip strategy in recovering OLB

**Fig 5:**
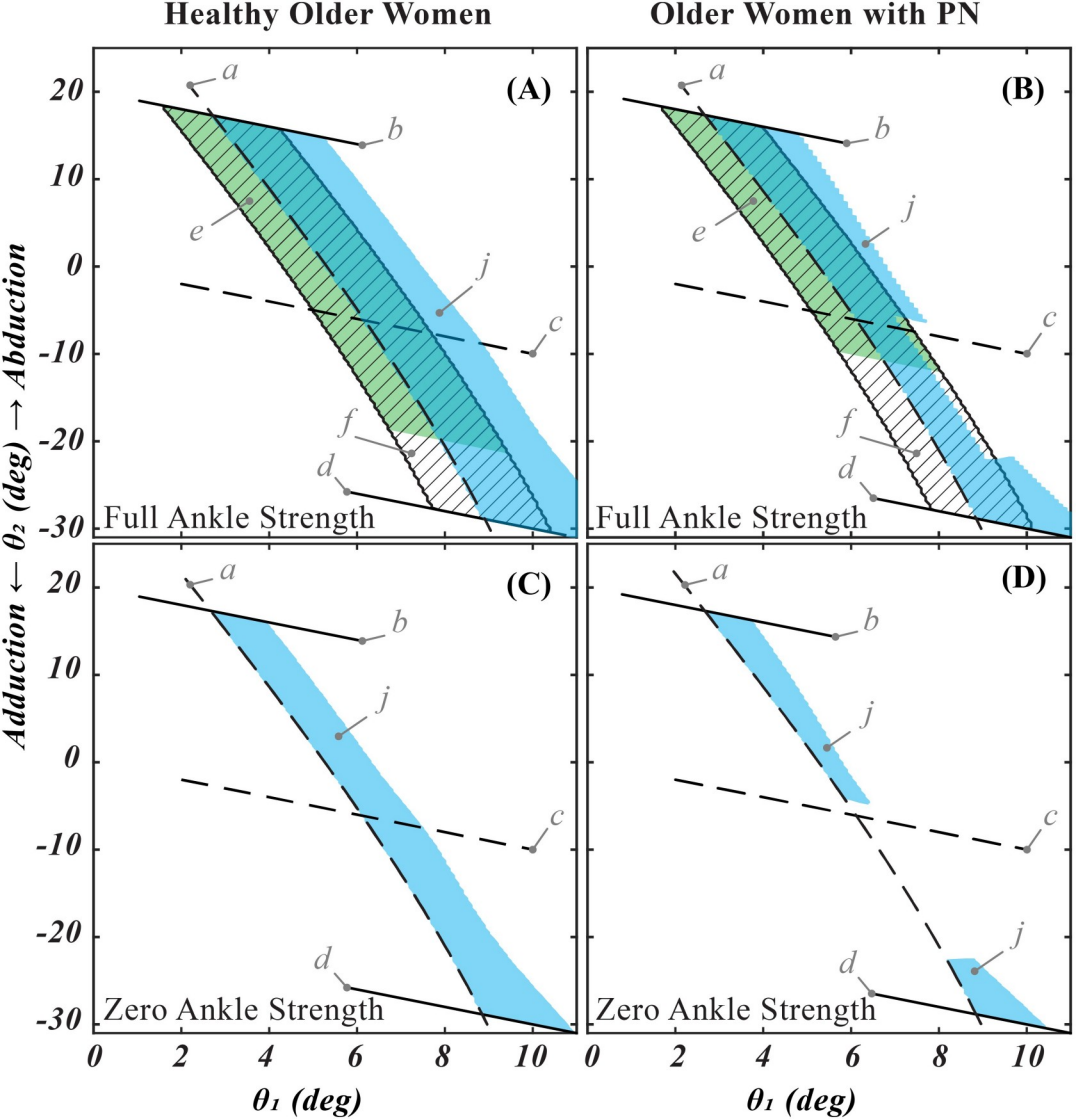
Graphical comparison of calculated active and net FBR with recoverable laterally perturbed initial quasistatic OLB states in healthy older women and older women with peripheral neuropathy. Letters a-d show the loci of quasistatic initial states similar to those shown in Fig 3. Letter e (green region) shows the active FBR, derived with only active hip abduction strength. Letter f (region with diagonal lines) shows net FBR derived with both active and passive hip abduction moment. Letter j (blue region) shows laterally perturbed initial quasistatic states which can be recovered by a strategy involving maximum acceleration of the center of mass. We can see that while for both groups the net FBR extends between lines b and d, weaker active hip abduction strength significantly ‘hollows out’ the recoverable states for the older women with PN (A and B). Repeating the simulations with zero ankle inversion / eversion strength collapses both active and net FBR to line a, and shows the same hollowing effect on the recoverable quasistatic states as the simulations with full ankle strength (C and D). Comparing the width of the FBR (top row) and the recoverable quasistatic states when considering no ankle strength (bottom row) shows that the effectiveness of the hip strategy in recovering OLB heavily depends on the maximum hip abduction strength. In the healthy older women, the hip strategy seems as effective as the ankle strategy.

All initial quasistatic states, where our simulations predict recovering OLB from an impending lateral fall was possible, are shown in Fig 5 (blue region). Despite the net FBR being similar between the healthy older women and older women with PN, a shorter active FBR (weaker hip abduction strength) in the latter led to a hollowing of the recoverable states. Comparing the width of the FBR (Fig 5, top row) and the recoverable quasistatic states when considering no ankle strength (Fig 5, bottom row) the effectiveness of the hip strategy in recovering OLB is seen to heavily depend on the maximum hip abduction strength. Indeed in the healthy older women, the hip strategy was as effective as the ankle strategy.

## 4. Discussion

### 4.1. What’s new?

We believe this is the first biomechanical study of OLB to investigate the effect of limited maximum hip abduction strength on quasistatic stance and balance recovery. Previous biomechanical studies have either: a) ignored the stance hip altogether in favor of a simpler single inverted pendulum model that only considered stance ankle and foot mechanics [8, 11], b) despite using a double inverted pendulum model, incorrectly assumed the center of mass of the body without the stance leg to lie in the same vertical plane as the stance hip during OLB [9, 10], or c) utilized more complicated multi-joint models to study dynamic tasks different than clinical OLB such as standing on one leg on a narrow ridge or the single stance leg phase of gait [22, 37]. The present methods provide an approach to begin to study what a ‘loss of balance’ looks like given the simplifying assumptions of the model. Yet even for this simple two-link model, the state-space for the full dynamic OLB is four dimensional (i.e., hip and ankle angles as well as angular velocities), making it difficult to visualize. So we resorted to assuming initial quasistatic OLB states to ameliorate this difficulty and visualize the two-dimensional state spaces (for example, Fig 5).

### 4.2. Hip abduction moment demand during quasistatic OLB

The presence of the hip joint in the present model allows one to estimate the hip abduction moment demand of OLB. For a level pelvis this ranged from 50% to 106% of the maximum hip abduction strength depending on the population group being studied (Results 4.1). These results are in agreement with Inman’s and Charnley’s calculations for standing with a level pelvis [19, 20]. So, we conclude the hip abduction moment demand in OLB is sufficiently high to warrant the use of a model including a hip joint.

### 4.3. Investigating OLB with the FBR

FBR is essentially an extension of the FBOS criterion for quasistatic OLB (see Introduction) to account for limited hip abduction strength. The lateral and medial margins of the FBOS can be seen as the two lines parallel to the T_S_ = 0 locus of points (Fig 2). On the other hand, if the patient does not have enough hip abduction strength, then the active FBR will not extend all the way between the lines indicating a nominal 20º increase and decrease in pelvic inclination angle. Since the intrinsic noise in physiological feedback loops and force generation within the muscles causes random variations in the OLB states (for example [38]), an important insight is that a reduction in FBR size makes maintaining quasistatic OLB a more difficult task. Factors increasing the random motion of the OLB states, such as loss of sensory function or recruiting higher muscle forces [39], increases the random motion, thereby effectively shrinking the FBR. If one’s quasistatic state is inside and away from the boundaries of the FBR, then fine adjustments of the center of pressure (COP) and maintaining a nearly constant hip abduction moment can control the state without the need for any dynamic maneuvers. However, if the quasistatic state nears an FBR boundary, that is an indication that the strength defining that boundary is nearly saturated. In the case of individuals with strong hip abductors, the only FBR boundaries encountered are the lines defined by the margins of FBOS (Fig 2). In that case, the simpler method of investigating COP movements within the FBOS would be enough for interpreting the results of the OLB test. This is true for studies of OLB with healthy young subjects [8]. On the other hand, loss of hip strength due to aging or disease can make the maximum hip abduction strength an active constraint during OLB. This is clearly the case if we neglect the passive hip abduction moment and only consider active FBR (Fig 2). Unlike the FBOS boundaries that can be avoided by rapid hip moment generation [12, 22], nearing a maximum hip abduction strength boundary essentially leads to the end of a OLB trial unless the subject violates the assumption of no lateral bending of the trunk. If we consider the contribution of passive hip tissue to the abduction moment, then only a severe loss of strength would make the maximum hip abduction strength an active constraint (Fig 4).

### 4.4. Passive tissue contribution to the hip abduction moment

Decreasing the pelvic inclination angle in OLB *(θ*_*1*_ *+ θ*_*2*_*)* increases hip abduction moment demand on a cosine curve, while it increases the passive tissue moment exponentially. The reverse is also true for an increase in the pelvic inclination angle. The positive Trendelenburg test of gluteal function is an attestation to this interaction; some patients with severe gluteal damage stand on one leg with a marked decrease in pelvic inclination angle [5]. We believe a knowledge gap is that there are currently no studies reporting direct measurement of the abduction moment generated about the hip by the passive tissues as one varies adduction angle. As already explained (see Methods, 3.2) one of two relevant studies that were used for estimation of the passive tissue hip abduction moment used cadaver specimens with all tissues but the hip capsular ligaments removed. While these results underestimated curves for the possible contribution of the passive tissue hip abduction moment during quasistatic OLB, they still proved sufficient to compensate for the loss of strength due to aging and PN in women. This can be seen by comparing the active and net FBR in Figs 2 and 3. However, in case of further loss of hip abduction strength we showed that, while accounting for the passive tissue hip abduction moment enlarged the net FBR compared to the active FBR, it was not enough for older men with PN and led to a dividing of the net FBR in the middle close to the level pelvic inclination angle (Fig 4). The lower part corresponds to a positive Trendelenburg sign, while the upper part corresponds to a negative Trendelenburg sign in which patients essentially reduce the hip abduction moment demand by bending their torso in the ipsilateral direction [5].

### 4.5. Active FBR, Net FBR, and loss of balance recoverability

The results show that maintaining quasistatic balance and recovering OLB are not the same, yet both are important for a successful OLB trial. While maintaining quasistatic balance, one’s OLB states (Fig 1C) have to remain within the boundaries of the FBR. Due to the abduction moment from passive tissues acting about the hip joint, the net FBR for most people only has two active boundaries: these correspond to the lateral and medial margins of the FBOS. However, when the quasistatic state approaches one of these boundaries or even crosses them, the subject can resort to a dynamic shear force strategy [12] to recover the OLB state back to a quasistatic state inside the net FBR. In our simple model with only the stance hip and ankle joints, the rest of the body was considered rigid. Then in case of recovering from an impending ipsilateral fall, the shear force strategy reduces to a rapid increase in hip abduction moment that leads to lifting the contralateral hip (increasing pelvic inclination angle). So the rate of torque development (RTD) is the factor that determines the effectiveness of this hip strategy for recovering OLB. Given that maximum RTD and maximum active strength are positively correlated [36], the effectiveness of a hip strategy for recovering OLB is governed by the active hip abduction strength. The result of our simulations (Fig 5) confirm this reasoning. Comparing the net FBR for older healthy women (Fig 5A) and older women with PN (Fig 5B) shows no difference except for a narrowing of the region due to weaker ankle strength in the latter group; however the active FBR is clearly shorter due to decreasing maximum hip abduction strength. This then leads to the difference between recoverable quasistatic OLB states for the two groups. For healthy older women, addition of the hip strategy in calculating the recoverable states evenly extends the boundary corresponding to the ipsilateral margin of FBOS. However for older women with PN, one can see that this extension is hollowed just above where the active FBR ends. Repeating the simulations with no ankle strength (Fig 5C and 5D) shows the same effect, leading us to believe the difference in hip abduction strength was the driving factor for this difference. It also provides a visualization aid for comparing the effectiveness of the hip strategy and the ankle strategy in controlling OLB. So, while the net FBR corresponds to the effect of ankle and hip strength on quasistatic OLB, the active FBR can be used as a measure of OLB recoverability.

### 4.6. What can clinicians learn from this study?

Clinical OLB is quasistatic; in some cases to ensure the patient’s safety, the examiner might even stop the trial if rapid movements of the torso and arms are observed. So the patient’s OLB states are quasistatic and therefore have to lie for the most part inside the FBR. Our results show that even including an underestimation of passive hip abduction moment in visualizing the net FBR, limits the difference between healthy young and older adults to the difference in effective ankle strength (FBOS width). So, unless the population being studied has a severe loss of hip strength, hip abduction strength should not play a role in differentiating patients during quasistatic OLB. So the current test of clinical OLB is more sensitive to ankle strength, sensory feedback, and coordination than to changes in hip abduction strength. These results help explain why a timed Trendelenburg sign test performs better than the conventional test [5]; sustained effort in the hip abductor muscles during OLB leads to fatigue and reduction of effective maximum strength over time according to Rohmert’s curve [40]. If the maximum hip abduction strength is close to the critical value where a splitting of the net FBR occurs, then a Trendelenburg sign will be observed after fatigue. This also explain why even a 50% experimental reduction in maximum hip abduction strength by ultrasound nerve blocking did not evoke a Trendelenburg sign in a group of nine healthy young men [41]. We therefore suggest a modified Trendelenburg test should be added to the clinical OLB test. The patient could be asked to stand on one leg with a slightly raised contralateral hip while being allowed to use small occasional finger touches to a nearby support to help with the coordination. This test will increase the contribution of the abductor muscles to providing the demand of the OLB as shown by EMG measurements [19, 21]. Since muscles are more involved in meeting the abduction moment demand of OLB, they will fatigue easier, which then allows the measured time to be used as a proxy for maximum hip abduction strength measurement without the need for dynamometers. Such a measure can better capture the capacity of the individual to be able to use a hip strategy in order to avoid a lateral fall from OLB compared to the quasistatic OLB test.

### 4.7. Strengths and limitations

In this study the simplest model of OLB was used that could allow one to include hip strength in the analysis. The simplicity of the model helps one characterize the effect of ankle and hip strength on quasistatic OLB states in the form of the net FBR, and a method for comparing the effectiveness of the hip and ankle strategies. These results are an upper bound estimation for what is physically possible since sensory feedback signals were assumed to be perfect with no delay across all population groups. For a patient population such as those with peripheral neuropathy, these upper bound estimates are greatly overestimating the OLB capacity. In spite of this, our approach is still successful in pointing out that while ankle sensorimotor function is the focus for these patients, their hip strength is also automatically putting them at considerable risk for falls, a point that is currently usually neglected.

Amongst the most important limitations of this study are modeling errors and extracting anthropometric and capacity related parameters of the model from published mean values in the literature. *First*, we used a double inverted pendulum model for all our analyses. This required considering the arms, head, non-stance leg, and particularly the spine to be always kept rigid. In addition the stance foot was assumed to be stationary while in a typical OLB in the daily life, one might even shuffle the stance foot medially or laterally to help control balance. Such a simplification was a compromise for finding the simplest model that could still capture the importance of the stance hip joint during OLB. In S4 Text the sensitivity of our findings to some of these assumption is given. *Second*, we used published mean muscle capacity data from the literature and scaled them to the height and weight of our 50^th^ percentile man model. Unfortunately, this approach neglects the between-subject differences in mass distribution, anthropometry, strengths and ROM. In spite of these assumptions the model still provides useful insights. *Third*, our estimation of the passive hip abduction moment was based on the two quantitative articles that we could find in the literature. While our model underestimates the passive hip abduction moment it is still capable of showing the substantial difference that it makes by comparing the shapes of the active and net FBR. This underlines the importance of obtaining accurate measurements of the passive hip abduction moment close to the end of adduction range of motion. *Finally*, the assumption that the maintenance of OLB is accomplished by movements completely contained within the frontal plane may not always be valid. For example, at the stance hip, one can redirect torso momentum in the frontal plane to the sagittal plane by engaging the hip internal or external rotator muscles. Despite these limitations we believe the model provides useful insights into the clinical test of OLB.

## 5. Conclusions

1. The FBR is a useful tool for capture the different states in which the strength requirements are met for quasistatic OLB.
2. Comparison of the active FBR and the net FBR revealed that the contribution of passive tissues to the net hip abduction moment can mask moderate deficits in hip abduction strength in a clinical OLB test.
3. In contrast, quasistatic OLB states that could be recovered from a lateral fall greatly depended upon adequate maximum hip abduction strength, better captured by the active FBR.
4. We therefore suggest that, in the clinic, adding a follow-up OLB test with a slightly raised pelvis is a simple way to check for adequate hip abductor muscle strength.

## Supporting information

S1 Text Scaling of ankle and hip strengths to the double inverted pendulum model. (DOCX)

S2 Text Deriving the equations of motion for the double inverted pendulum model. (DOCX)

S3 Text Simulation of the planar movements of a double inverted pendulum model with range of motion constraints. (DOCX)

S4 Text Predicted sensitivity of active FBR and hip abduction moment to medio-lateral bending of the trunk and movements of the contralateral leg in the frontal plane during OLB. (DOCX)
