# Supplementary material for: On the importance of the hip abductors during a clinical one legged balance test: A theoretical study
Source: PLoS One. 2020 Nov 13;15(11):e0242454. doi: 10.1371/journal.pone.0242454 (PMC7665826; doi:10.1371/journal.pone.0242454)
Supplement: S1 Text — (DOCX) [file pone.0242454.s001.docx]

S1 Text: Scaling of ankle and hip strengths to the double inverted pendulum model

There are few reports with enough anthropometric and mass distribution information, which is needed for creating the double inverted pendulum model (1,2); most of these are of younger men. Therefore, we could not make a different model for each of the population of men and women in our study (healthy younger adults, healthy older adults, and older patients with peripheral neuropathy). Instead, we resorted to scaling of the strength data. In this document we will first demonstrate the procedure for scaling the ankle and hip strengths, followed by a table of strength values used for the simulations of the model.

Classic studies by Sepic *et al.* and Cahalan *et al.* report ankle inversion / eversion and hip abduction / adduction strengths for healthy younger and older adults respectively (3,4). We normalized these values by the reported mean mass and height of the subjects in each group of men and women separately. Then the normalized strength values were scaled to the parameters of the model by multiplying them by the mass and height of the mid-size man (1) in our model (mass = 81.4 Kg, height = 1.78 m). The normalized ankle and hip strength values for the older patients with peripheral neuropathy were extracted from a paper by Allet *et al.* which also report normalized strengths for healthy older adults (5). A comparison of the data of healthy older adults between Sepic and Allet and Cahalan and Allet revealed clear differences, suggesting different methodology in measurements. We chose to use the Sepic and Cahalan values for the healthy older adults; to account for the difference in methodology for the strength data of older patients with peripheral neuropathy, the following formula was used to arrive at the adjusted values for the normalized strength (Norm. = normalized, Str. = strength, PN = peripheral neuropathy).

$Norm.Str. PN={Norm. Str. Healthy}_{Cahalan/Sepic}\times\frac{{Norm. Str. PN}_{Allet}}{{Norm.Str.Healthy}_{Allet}}$

The following table shows the original data, mass and height data from each report, and final scaled strengths applied to the model in this study.

Table 1- Original and scaled ankle and hip strength data used in the simulation of the study

| Healthy Younger Men | Mean Strength (N.m) | Mean Mass (Kg) | Mean Height (m) | Normalized Strength (N.m/N.m) | Model Scaled Strength (N.m) |
| --- | --- | --- | --- | --- | --- |
| Ankle Inversion | 21.4 | 82.2 | 1.77 | 0.015 | 21.3 |
| Ankle Eversion | 15.3 |  |  | 0.011 | 15.2 |
| Hip Abduction | 108 | 73.5 | 1.76 | 0.085 | 121 |
| Hip Adduction | 83 |  |  | 0.065 | 93 |
| Healthy Younger Women | |  |  |  |  |
| Ankle Inversion | 13.6 | 59.7 | 1.64 | 0.014 | 20.2 |
| Ankle Eversion | 10.6 |  |  | 0.011 | 15.9 |
| Hip Abduction | 72 | 58.6 | 1.67 | 0.075 | 106.6 |
| Hip Adduction | 58 |  |  | 0.060 | 85.9 |
| Healthy Older Men | |  |  |  |  |
| Ankle Inversion | 18 | 83 | 1.71 | 0.013 | 18.3 |
| Ankle Eversion | 14.1 |  |  | 0.010 | 14.4 |
| Hip Abduction | 90 | 80.5 | 1.76 | 0.065 | 92 |
| Hip Adduction | 77 |  |  | 0.055 | 78.7 |
| Healthy Older Women | |  |  |  |  |
| Ankle Inversion | 14 | 68.2 | 1.64 | 0.013 | 18.2 |
| Ankle Eversion | 10.1 |  |  | 0.009 | 13.1 |
| Hip Abduction | 55 | 64.9 | 1.64 | 0.053 | 74.9 |
| Hip Adduction | 49 |  |  | 0.047 | 66.7 |
| Older Men with PN | |  |  |  |  |
| Ankle Inversion |  | | | 0.008 | 11.4 |
| Ankle Eversion |  |  |  | 0.007 | 9.7 |
| Hip Abduction |  |  |  | 0.041 | 57.8 |
| Hip Adduction |  |  |  | 0.038 | 54 |
| Older Women with PN | |  |  |  |  |
| Ankle Inversion |  | | | 0.011 | 15.2 |
| Ankle Eversion |  |  |  | 0.008 | 11.9 |
| Hip Abduction |  |  |  | 0.045 | 64.2 |
| Hip Adduction |  |  |  | 0.031 | 44.5 |

**S1 Text References**:

1. Harry G. Armstrong Aerospace Medical Research Laboratory. Anthropometry and Mass Distribution for Human Analogues: Military Male Aviators. 1988;I.

2. Winter DA. Anthropometry. In: Biomechanics and Motor Control of Human Movement. fourth edi. John Wiley & Sons; 2009. p. 82–106.

3. Sepic SB, Murray MP, Mollinger LA, Spurr GB, Gardner GM. Strength and range of motion in the ankle in two age groups of men and women. Am J Phys Med. 1986;65(2):75–84.

4. Cahalan TD, Johnson ME, Liu S, Chao EY. Quantitative measurements of hip strength in different age groups. Clin Orthop Relat Res. 1989;(246):136–45.

5. Allet L, Kim H, Ashton-Miller JA, DeMott TK, Richardson JK. Frontal plane hip and ankle sensorimotor function, not age, predicts unipedal stance time. Muscle Nerve. 2012;45(4):578–85.
