# Supplementary material for: On the importance of the hip abductors during a clinical one legged balance test: A theoretical study
Source: PLoS One. 2020 Nov 13;15(11):e0242454. doi: 10.1371/journal.pone.0242454 (PMC7665826; doi:10.1371/journal.pone.0242454)
Supplement: S2 Text — (DOCX) [file pone.0242454.s002.docx]

**S2 Text: Deriving the equations of motion for the double inverted pendulum model**

***1. Kinematics of the double inverted pendulum model***

| 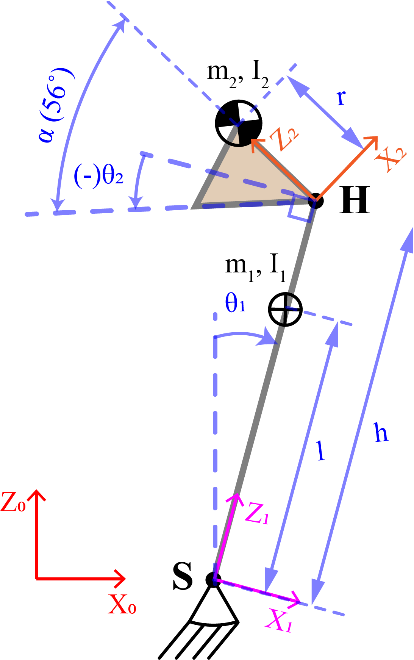 |
| --- |

$$\vec{r}_{m_{1}}=l \hat{k}_{1}$$

$$\vec{r}_{H}=h \hat{k}_{1}$$

$$\vec{r}_{m_{2}}=\vec{r}_{H}+r \hat{k}_{2}=h \hat{k}_{1}+r \hat{k}_{2}$$

$$\vec{v}_{m_{1}}=\dot{\theta}_{1} \hat{j}\times l \hat{k}_{1}$$

$$\vec{v}_{m_{1}}={l\dot{\theta}}_{1} \hat{i}_{1}$$

$$\vec{v}_{H}={h\dot{\theta}}_{1} \hat{i}_{1}$$

$$\vec{v}_{m_{2}}=\vec{v}_{H}+\left( \dot{\theta}_{1}+\dot{\theta}_{2} \right)\hat{j}\times r \hat{k}_{2}$$

$$\vec{v}_{m_{2}}=\left( {h\dot{\theta}}_{1} \right) \hat{i}_{1}+r\left( \dot{\theta}_{1}+\dot{\theta}_{2} \right) \hat{i}_{2}$$

$$\vec{a}_{m_{1}}=\ddot{\theta}_{1} \hat{j}\times l \hat{k}_{1}+\dot{\theta}_{1} \hat{j}\times(\dot{\theta}_{1}\hat{j}\times l \hat{k}_{1})$$

$\vec{a}_{m_{1}}={l\ddot{\theta}}_{1} \hat{i}_{1}-l{\dot{\theta}_{1}}^{2} \hat{k}_{1}$,

| 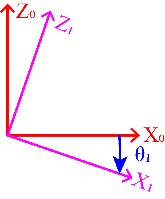 |
| --- |
| 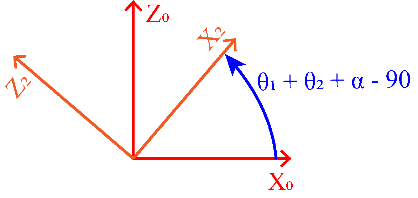 |

$\vec{a}_{m_{1}}=\left\{ \left. \begin{matrix} {l\ddot{\theta}}_{1} \\ 0 \\ -l{\dot{\theta}_{1}}^{2} \end{matrix} \right\} \right._{X_{1}Y_{1}}=\left\{ \left. \begin{matrix} {l\ddot{\theta}}_{1}\cos(\theta_{1})-l{\dot{\theta}_{1}}^{2}\sin(\theta_{1}) \\ 0 \\ {-l\ddot{\theta}}_{1}\sin(\theta_{1})-l{\dot{\theta}_{1}}^{2}\cos(\theta_{1}) \end{matrix} \right\} \right._{X_{0}Y_{0}}$,

$$\vec{a}_{H}={h\ddot{\theta}}_{1}\hat{i}_{1}-h{\dot{\theta}_{1}}^{2} \hat{k}_{1}$$

$$\vec{a}_{m_{2}}=\vec{a}_{H}+\left( \ddot{\theta}_{1}+\ddot{\theta}_{2} \right)\hat{j}\times r \hat{k}_{2}+\left( \dot{\theta}_{1}+\dot{\theta}_{2} \right)\hat{j}\times\left( \left( \dot{\theta}_{1}+\dot{\theta}_{2} \right)\hat{j}\times r \hat{k}_{2} \right)$$

$$\vec{a}_{m_{2}}=\vec{a}_{H}+r\left( \ddot{\theta}_{1}+\ddot{\theta}_{2} \right) \hat{i}_{2}-r\left( \dot{\theta}_{1}+\dot{\theta}_{2} \right)^{2} \hat{k}_{2}$$

$$\vec{a}_{m_{2}}=\left\{ \left. \begin{matrix} {h\ddot{\theta}}_{1}\cos(\theta_{1})-h{\dot{\theta}_{1}}^{2}\sin(\theta_{1}) \\ 0 \\ {-h\ddot{\theta}}_{1}\sin(\theta_{1})-h{\dot{\theta}_{1}}^{2}\cos(\theta_{1}) \end{matrix} \right\} \right._{X_{0}Y_{0}}+\left\{ \left. \begin{matrix} r\left( \ddot{\theta}_{1}+\ddot{\theta}_{2} \right) \\ 0 \\ -r\left( \dot{\theta}_{1}+\dot{\theta}_{2} \right)^{2} \end{matrix} \right\} \right._{X_{2}Y_{2}}$$

$$\vec{a}_{m_{2}}=\left\{ \left. \begin{matrix} {h\ddot{\theta}}_{1}\cos(\theta_{1})-h{\dot{\theta}_{1}}^{2}\sin(\theta_{1}) \\ 0 \\ {-h\ddot{\theta}}_{1}\sin(\theta_{1})-h{\dot{\theta}_{1}}^{2}\cos(\theta_{1}) \end{matrix} \right\} \right._{X_{0}Y_{0}}+\left\{ \left. \begin{matrix} r\left( \ddot{\theta}_{1}+\ddot{\theta}_{2} \right)\sin(\theta_{1}+\theta_{2}+\alpha)+r\left( \dot{\theta}_{1}+\dot{\theta}_{2} \right)^{2}\cos(\theta_{1}+\theta_{2}+\alpha) \\ 0 \\ r\left( \ddot{\theta}_{1}+\ddot{\theta}_{2} \right)\cos(\theta_{1}+\theta_{2}+\alpha)-r\left( \dot{\theta}_{1}+\dot{\theta}_{2} \right)^{2}\sin(\theta_{1}+\theta_{2}+\alpha) \end{matrix} \right\} \right._{X_{0}Y_{0}}$$

2. Planar Dynamics of the double inverted pendulum model

| 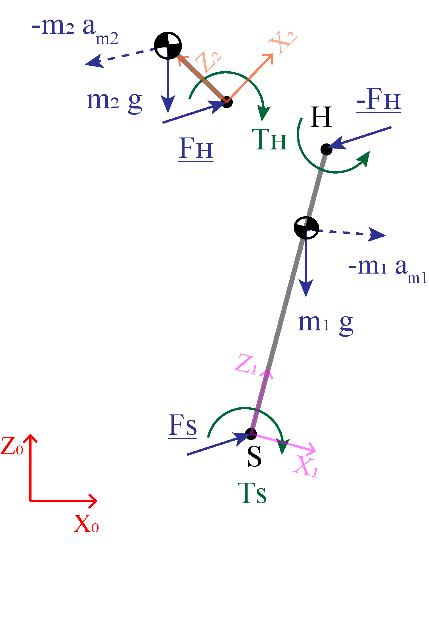 |
| --- |

$$\vec{g}=\left\{ \left. \begin{matrix} 0 \\ 0 \\ -g \end{matrix} \right\} \right._{X_{0}Y_{0}}$$

*Stance Leg (SL):*

$\sum\vec{F}=\vec{F}_{S}+m_{1}\left( \vec{g}-\vec{a}_{m_{1}} \right)-\vec{F}_{H}=0\leftrightarrow$,

$F_{Sx}=F_{Hx}+m_{1}a_{m_{1}x}\to$,

$F_{Sx}=F_{Hx}+m_{1}\left( {l\ddot{\theta}}_{1}\cos\left( \theta_{1} \right)-l{\dot{\theta}_{1}}^{2}\sin\left( \theta_{1} \right) \right)\to$,

$F_{Sx}=F_{Hx}+m_{1}{l\ddot{\theta}}_{1}\cos\left( \theta_{1} \right)-m_{1}l{\dot{\theta}_{1}}^{2}\sin\left( \theta_{1} \right)$,

Inverse Dynamics Equation 1

$F_{Sz}=F_{Hz}+m_{1}\left( a_{m_{1}z}+g \right)\to$,

$F_{Sz}=F_{Hz}+m_{1}\left( {-l\ddot{\theta}}_{1}\sin(\theta_{1})-l{\dot{\theta}_{1}}^{2}\cos(\theta_{1})+g \right)\to$,

$F_{Sz}=m_{1}g+F_{Hz}-m_{1}{l\ddot{\theta}}_{1}\sin(\theta_{1})-m_{1}l{\dot{\theta}_{1}}^{2}\cos(\theta_{1})$,

Inverse Dynamics Equation 2

$\sum\vec{M}_{m_{1}}=\left( T_{S}-T_{H} \right) \hat{j}-l \hat{k}_{1}\times\vec{F}_{S}-\left( h-l \right)\hat{k}_{1}\times\vec{F}_{H}=I_{1}\ddot{\theta}_{1} \hat{j} \leftrightarrow$,

$\left( T_{S}-T_{H} \right)-F_{Sx}l\cos\left( \theta_{1} \right)+F_{Sz}l\sin\left( \theta_{1} \right)-F_{Hx}\left( h-l \right)\cos\left( \theta_{1} \right)+F_{Hz}\left( h-l \right)\sin\left( \theta_{1} \right)=I_{1}\ddot{\theta}_{1}$,

Inverse Dynamics Equation 3

*Rest of the Body (RB):*

$\sum\vec{F}=\vec{F}_{H}+m_{2}\left( \vec{g}-\vec{a}_{m_{2}} \right)=0 \leftrightarrow$,

$F_{Hx}=m_{2}a_{m_{2}x}\to$,

$F_{Hx}=m_{2}{h\ddot{\theta}}_{1}\cos\left( \theta_{1} \right)-m_{2}h{\dot{\theta}_{1}}^{2}\sin\left( \theta_{1} \right)+m_{2}r\left( \ddot{\theta}_{1}+\ddot{\theta}_{2} \right)\sin(\theta_{1}+\theta_{2}+\alpha)+m_{2}r\left( \dot{\theta}_{1}+\dot{\theta}_{2} \right)^{2}\cos(\theta_{1}+\theta_{2}+\alpha)$,

Inverse Dynamics Equation 4

$F_{Hz}=m_{2}g+m_{2}a_{m_{2}z}\to$,

$F_{Hz}=m_{2}g-m_{2}{h\ddot{\theta}}_{1}\sin\left( \theta_{1} \right)-m_{2}h{\dot{\theta}_{1}}^{2}\cos\left( \theta_{1} \right)+m_{2}r\left( \ddot{\theta}_{1}+\ddot{\theta}_{2} \right)\cos(\theta_{1}+\theta_{2}+\alpha)-m_{2}r\left( \dot{\theta}_{1}+\dot{\theta}_{2} \right)^{2}\sin(\theta_{1}+\theta_{2}+\alpha)$,

Inverse Dynamics Equation 5

$\sum\vec{M}_{m_{2}}=T_{H} \hat{j}-r \hat{k}_{2}\times\vec{F}_{H}=I_{2}(\ddot{\theta}_{1}+\ddot{\theta}_{2})\hat{j}\to$,

$T_{H}-F_{Hx}r\sin\left( \theta_{1}+\theta_{2}+\alpha\right)-F_{Hz}r\cos\left( \theta_{1}+\theta_{2}+\alpha\right)=I_{2}(\ddot{\theta}_{1}+\ddot{\theta}_{2})$,

Inverse Dynamics Equation 6

Replace F_S_ values into Inverse Dynamics Equation 3:

$\left( T_{S}-T_{H} \right)-\left( F_{Hx}+m_{1}{l\ddot{\theta}}_{1}\cos\left( \theta_{1} \right)-m_{1}l{\dot{\theta}_{1}}^{2}\sin\left( \theta_{1} \right) \right)l\cos\left( \theta_{1} \right)+\left( m_{1}g+F_{Hz}-m_{1}{l\ddot{\theta}}_{1}\sin\left( \theta_{1} \right)-m_{1}l{\dot{\theta}_{1}}^{2}\cos\left( \theta_{1} \right) \right)l\sin\left( \theta_{1} \right)-F_{Hx}\left( h-l \right)\cos\left( \theta_{1} \right)+F_{Hz}\left( h-l \right)\sin\left( \theta_{1} \right)=I_{1}\ddot{\theta}_{1}$,

And now replace F_H_ values in the above equation:

$\left( T_{S}-T_{H} \right)-\left( m_{1}{l\ddot{\theta}}_{1}\cos\left( \theta_{1} \right)-m_{1}l{\dot{\theta}_{1}}^{2}\sin\left( \theta_{1} \right) \right)l\cos\left( \theta_{1} \right)+\left( m_{1}g-m_{1}{l\ddot{\theta}}_{1}\sin\left( \theta_{1} \right)-m_{1}l{\dot{\theta}_{1}}^{2}\cos\left( \theta_{1} \right) \right)l\sin\left( \theta_{1} \right)-\left( m_{2}{h\ddot{\theta}}_{1}\cos\left( \theta_{1} \right)-m_{2}h{\dot{\theta}_{1}}^{2}\sin\left( \theta_{1} \right)+m_{2}r\left( \ddot{\theta}_{1}+\ddot{\theta}_{2} \right)\sin\left( \theta_{1}+\theta_{2}+\alpha\right)+m_{2}r\left( \dot{\theta}_{1}+\dot{\theta}_{2} \right)^{2}\cos\left( \theta_{1}+\theta_{2}+\alpha\right) \right)h\cos\left( \theta_{1} \right)+\left( m_{2}g-m_{2}{h\ddot{\theta}}_{1}\sin\left( \theta_{1} \right)-m_{2}h{\dot{\theta}_{1}}^{2}\cos\left( \theta_{1} \right)+m_{2}r\left( \ddot{\theta}_{1}+\ddot{\theta}_{2} \right)\cos\left( \theta_{1}+\theta_{2}+\alpha\right)-m_{2}r\left( \dot{\theta}_{1}+\dot{\theta}_{2} \right)^{2}\sin\left( \theta_{1}+\theta_{2}+\alpha\right) \right)h\sin\left( \theta_{1} \right)=I_{1}\ddot{\theta}_{1}\to$,

$\left( T_{S}-T_{H} \right)-m_{1}l^{2}{\cos\left( \theta_{1} \right)}^{2}\ddot{\theta}_{1}+m_{1}l^{2}\sin\left( \theta_{1} \right)\cos\left( \theta_{1} \right){\dot{\theta}_{1}}^{2}+m_{1}gl\sin\left( \theta_{1} \right)-m_{1}l^{2}{\sin\left( \theta_{1} \right)}^{2}\ddot{\theta}_{1}-m_{1}l^{2}\sin\left( \theta_{1} \right)\cos\left( \theta_{1} \right){\dot{\theta}_{1}}^{2}-m_{2}h^{2}{\cos\left( \theta_{1} \right)}^{2}\ddot{\theta}_{1}+m_{2}h^{2}\sin\left( \theta_{1} \right)\cos\left( \theta_{1} \right){\dot{\theta}_{1}}^{2}-m_{2}rh\sin\left( \theta_{1}+\theta_{2}+\alpha\right)\cos\left( \theta_{1} \right)\left( \ddot{\theta}_{1}+\ddot{\theta}_{2} \right)-m_{2}rh\cos\left( \theta_{1}+\theta_{2}+\alpha\right)\cos\left( \theta_{1} \right)\left( \dot{\theta}_{1}+\dot{\theta}_{2} \right)^{2}+m_{2}gh\sin\left( \theta_{1} \right)-m_{2}h^{2}{\sin\left( \theta_{1} \right)}^{2}\ddot{\theta}_{1}-m_{2}h^{2}\sin\left( \theta_{1} \right)\cos\left( \theta_{1} \right){\dot{\theta}_{1}}^{2}+m_{2}rh\cos\left( \theta_{1}+\theta_{2}+\alpha\right)\sin\left( \theta_{1} \right)\left( \ddot{\theta}_{1}+\ddot{\theta}_{2} \right)-m_{2}rh\sin\left( \theta_{1}+\theta_{2}+\alpha\right)\sin\left( \theta_{1} \right)\left( \dot{\theta}_{1}+\dot{\theta}_{2} \right)^{2}=I_{1}\ddot{\theta}_{1}\to$,

$T_{S}-T_{H}+\left( m_{1}l+m_{2}h \right)g\sin\left( \theta_{1} \right)-m_{2}rh\sin\left( \theta_{2}+\alpha\right)\left( \ddot{\theta}_{1}+\ddot{\theta}_{2} \right)-m_{2}rh\cos\left( \theta_{2}+\alpha\right)\left( \dot{\theta}_{1}+\dot{\theta}_{2} \right)^{2}=\left( I_{1}+m_{1}l^{2}+m_{2}h^{2} \right)\ddot{\theta}_{1}$,

Double Inverted Pendulum Equation 1

Similarly replace F_H_ values into Inverse Dynamics Equation 6:

$T_{H}-m_{2}hr\sin\left( \theta_{1}+\theta_{2}+\alpha\right)\cos\left( \theta_{1} \right)\ddot{\theta}_{1}+m_{2}hr\sin\left( \theta_{1}+\theta_{2}+\alpha\right)\sin\left( \theta_{1} \right){\dot{\theta}_{1}}^{2}-m_{2}r^{2}{\sin\left( \theta_{1}+\theta_{2}+\alpha\right)}^{2}\left( \ddot{\theta}_{1}+\ddot{\theta}_{2} \right)-m_{2}r^{2}\sin\left( \theta_{1}+\theta_{2}+\alpha\right)\cos\left( \theta_{1}+\theta_{2}+\alpha\right)\left( \dot{\theta}_{1}+\dot{\theta}_{2} \right)^{2}-m_{2}gr\cos\left( \theta_{1}+\theta_{2}+\alpha\right)+m_{2}hr\sin\left( \theta_{1} \right)\cos\left( \theta_{1}+\theta_{2}+\alpha\right)\ddot{\theta}_{1}+m_{2}hr\cos\left( \theta_{1}+\theta_{2}+\alpha\right)\cos\left( \theta_{1} \right){\dot{\theta}_{1}}^{2}-m_{2}r^{2}{\cos\left( \theta_{1}+\theta_{2}+\alpha\right)}^{2}\left( \ddot{\theta}_{1}+\ddot{\theta}_{2} \right)+m_{2}r^{2}\sin\left( \theta_{1}+\theta_{2}+\alpha\right)\cos\left( \theta_{1}+\theta_{2}+\alpha\right)\left( \dot{\theta}_{1}+\dot{\theta}_{2} \right)^{2}=I_{2}(\ddot{\theta}_{1}+\ddot{\theta}_{2})\to$,

$T_{H}-m_{2}gr\cos\left( \theta_{1}+\theta_{2}+\alpha\right)-m_{2}hr\sin\left( \theta_{2}+\alpha\right)\ddot{\theta}_{1}+m_{2}hr\cos\left( \theta_{2}+\alpha\right){\dot{\theta}_{1}}^{2}=\left( I_{2}+m_{2}r^{2} \right)\left( \ddot{\theta}_{1}+\ddot{\theta}_{2} \right)$,

Double Inverted Pendulum Equation 2

The above equations then can be combined together in a matrix equation that results in the ***equations of motion for the double inverted pendulum***:

$$\left[ MM \right]\left\{ \begin{matrix} \ddot{\theta}_{1} \\ \ddot{\theta}_{2} \end{matrix} \right\}=\left[ BB \right]\left\{ \begin{matrix} T_{S} \\ T_{H} \end{matrix} \right\}+\left\{ CC \right\}$$

$$\left\{ \begin{matrix} \ddot{\theta}_{1} \\ \ddot{\theta}_{2} \end{matrix} \right\}=\left[ MM \right]^{-1}\left[ BB \right]\left\{ \begin{matrix} T_{S} \\ T_{H} \end{matrix} \right\}+\left[ MM \right]^{-1}\left\{ CC \right\}$$

Double Inverted Pendulum Equation 3

where,

$\left[ MM \right]=\left[ \begin{matrix} I_{1}+m_{1}l^{2}+m_{2}h^{2}+m_{2}rh\sin\left( \theta_{2}+\alpha\right) & m_{2}rh\sin\left( \theta_{2}+\alpha\right) \\ I_{2}+m_{2}r^{2}+m_{2}rh\sin\left( \theta_{2}+\alpha\right) & \left( I_{2}+m_{2}r^{2} \right) \end{matrix} \right]$,

$\left[ BB \right]=\left[ \begin{matrix} 1 & -1 \\ 0 & 1 \end{matrix} \right]$,

$\left\{ CC \right\}=\left\{ \begin{matrix} \left( m_{1}l+m_{2}h \right)g\sin\left( \theta_{1} \right)-m_{2}rh\cos\left( \theta_{2}+\alpha\right)\left( \dot{\theta}_{1}+\dot{\theta}_{2} \right)^{2} \\ -m_{2}gr\cos\left( \theta_{1}+\theta_{2}+\alpha\right)+m_{2}rh\cos\left( \theta_{2}+\alpha\right){\dot{\theta}_{1}}^{2} \end{matrix} \right\}$,

***3. Equilibrium equations for the double inverted pendulum***

Replacing angular velocity and acceleration terms with zero in the Double Inverted Pendulum Equations 1&2 simplifies them to the equilibrium equations for the double inverted pendulum model.

$T_{H}=T_{S}+\left( m_{1}l+m_{2}h \right)g\sin\left( \theta_{1} \right)$,

$T_{H}=m_{2}gr\cos\left( \theta_{1}+\theta_{2}+\alpha\right)$.

***4.*** ***Angular acceleration of the stance ankle-COM line:***

| 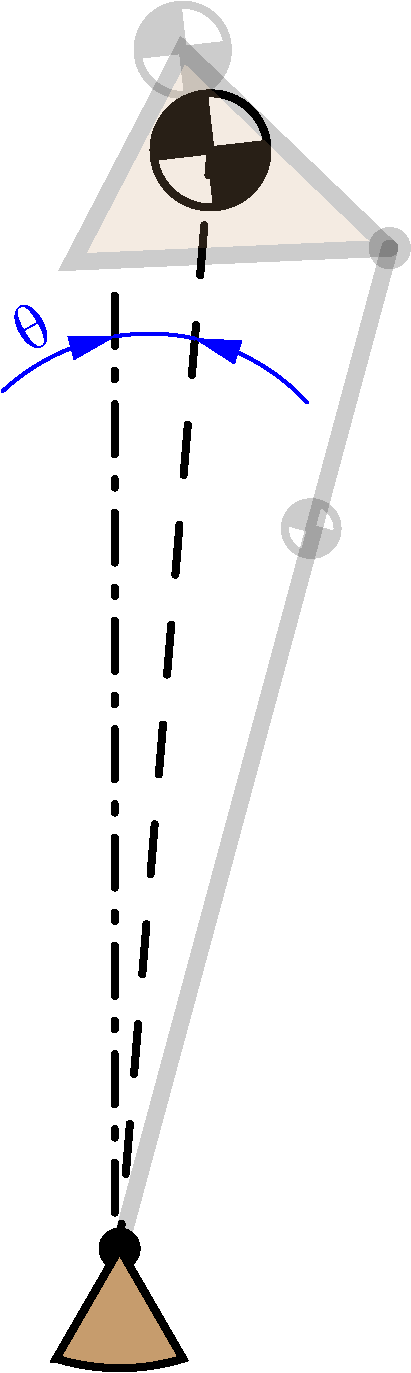 |
| --- |

Given ($\theta_{1},\theta_{2}$), function f calculates the tangent of the angle that the stance ankle-COM line makes with the vertical line ($\theta$).

$$\tan(\theta)=f(\theta_{1}, \theta_{2})\to\frac{d^{2}\left( \tan\left( \theta\right) \right)}{{dt}^{2}}=\frac{d^{2}\left( f\left( \theta_{1},\theta_{2} \right) \right)}{{dt}^{2}}$$

$$LHS 1: \frac{d(\tan(\theta))}{dt}=\left( 1+{\tan(\theta)}^{2} \right)\dot{\theta}=\left( 1+f^{2} \right)\dot{\theta}$$

$$LHS 2: \frac{d\left( \left( 1+{\tan\left( \theta\right)}^{2} \right)\dot{\theta} \right)}{dt}=\left( 1+f^{2} \right)\ddot{\theta}+2f\left( 1+f^{2} \right)\dot{\theta}^{2}$$

$$RHS 1: \frac{d\left( f\left( \theta_{1},\theta_{2} \right) \right)}{dt}=\left[ \begin{matrix} \frac{\partial f}{\partial\theta_{1}} & \frac{\partial f}{\partial\theta_{2}} \end{matrix} \right]\left\{ \begin{matrix} \dot{\theta}_{1} \\ \dot{\theta}_{2} \end{matrix} \right\}$$

$$RHS 2: \frac{d\left( \left[ \begin{matrix} \frac{\partial f}{\partial\theta_{1}} & \frac{\partial f}{\partial\theta_{2}} \end{matrix} \right]\left\{ \begin{matrix} \dot{\theta}_{1} \\ \dot{\theta}_{2} \end{matrix} \right\} \right)}{dt}=\left[ \begin{matrix} \frac{\partial f}{\partial\theta_{1}} & \frac{\partial f}{\partial\theta_{2}} \end{matrix} \right]\left\{ \begin{matrix} \ddot{\theta}_{1} \\ \ddot{\theta}_{2} \end{matrix} \right\}+\left\{ \begin{matrix} \dot{\theta}_{1} & \dot{\theta}_{2} \end{matrix} \right\}\left[ \begin{matrix} \frac{\partial^{2}f}{{\partial\theta_{1}}^{2}} & \frac{\partial^{2}f}{\partial\theta_{1}\partial\theta_{2}} \\ \frac{\partial^{2}f}{\partial\theta_{1}\partial\theta_{2}} & \frac{\partial^{2}f}{{\partial\theta_{2}}^{2}} \end{matrix} \right]\left\{ \begin{matrix} \dot{\theta}_{1} \\ \dot{\theta}_{2} \end{matrix} \right\}$$

Now we put LHS and RHS together again:

$$LHS 1=RHS 1 \to\left( 1+f^{2} \right)\dot{\theta}=\left[ \begin{matrix} \frac{\partial f}{\partial\theta_{1}} & \frac{\partial f}{\partial\theta_{2}} \end{matrix} \right]\left\{ \begin{matrix} \dot{\theta}_{1} \\ \dot{\theta}_{2} \end{matrix} \right\}\to\dot{\theta}=\frac{1}{\left( 1+f^{2} \right)}\left[ \begin{matrix} \frac{\partial f}{\partial\theta_{1}} & \frac{\partial f}{\partial\theta_{2}} \end{matrix} \right]\left\{ \begin{matrix} \dot{\theta}_{1} \\ \dot{\theta}_{2} \end{matrix} \right\}$$

$$\to LHS 2:\left( 1+f^{2} \right)\ddot{\theta}+2f\left( 1+f^{2} \right)\left( \frac{1}{\left( 1+f^{2} \right)}\left[ \begin{matrix} \frac{\partial f}{\partial\theta_{1}} & \frac{\partial f}{\partial\theta_{2}} \end{matrix} \right]\left\{ \begin{matrix} \dot{\theta}_{1} \\ \dot{\theta}_{2} \end{matrix} \right\} \right)^{2}$$

LHS 2 and RHS 2 are equal. Then:

$$\left( 1+f^{2} \right)\ddot{\theta}+\frac{2f}{\left( 1+f^{2} \right)}\left( \left[ \begin{matrix} \frac{\partial f}{\partial\theta_{1}} & \frac{\partial f}{\partial\theta_{2}} \end{matrix} \right]\left\{ \begin{matrix} \dot{\theta}_{1} \\ \dot{\theta}_{2} \end{matrix} \right\} \right)^{2}=\left[ \begin{matrix} \frac{\partial f}{\partial\theta_{1}} & \frac{\partial f}{\partial\theta_{2}} \end{matrix} \right]\left\{ \begin{matrix} \ddot{\theta}_{1} \\ \ddot{\theta}_{2} \end{matrix} \right\}+\left\{ \begin{matrix} \dot{\theta}_{1} & \dot{\theta}_{2} \end{matrix} \right\}\left[ \begin{matrix} \frac{\partial^{2}f}{{\partial\theta_{1}}^{2}} & \frac{\partial^{2}f}{\partial\theta_{1}\partial\theta_{2}} \\ \frac{\partial^{2}f}{\partial\theta_{1}\partial\theta_{2}} & \frac{\partial^{2}f}{{\partial\theta_{2}}^{2}} \end{matrix} \right]\left\{ \begin{matrix} \dot{\theta}_{1} \\ \dot{\theta}_{2} \end{matrix} \right\}$$

$$\ddot{\theta}=\frac{1}{\left( 1+f^{2} \right)}\left[ \begin{matrix} \frac{\partial f}{\partial\theta_{1}} & \frac{\partial f}{\partial\theta_{2}} \end{matrix} \right]\left\{ \begin{matrix} \ddot{\theta}_{1} \\ \ddot{\theta}_{2} \end{matrix} \right\}+\frac{1}{\left( 1+f^{2} \right)}\left( \left\{ \begin{matrix} \dot{\theta}_{1} & \dot{\theta}_{2} \end{matrix} \right\}\left[ \begin{matrix} \frac{\partial^{2}f}{{\partial\theta_{1}}^{2}} & \frac{\partial^{2}f}{\partial\theta_{1}\partial\theta_{2}} \\ \frac{\partial^{2}f}{\partial\theta_{1}\partial\theta_{2}} & \frac{\partial^{2}f}{{\partial\theta_{2}}^{2}} \end{matrix} \right]\left\{ \begin{matrix} \dot{\theta}_{1} \\ \dot{\theta}_{2} \end{matrix} \right\} \right)-\frac{2f}{\left( 1+f^{2} \right)^{3}}\left( \left\{ \begin{matrix} \dot{\theta}_{1} & \dot{\theta}_{2} \end{matrix} \right\}\left[ \begin{matrix} \left( \frac{\partial f}{\partial\theta_{1}} \right)^{2} & \frac{\partial f}{\partial\theta_{1}}\frac{\partial f}{\partial\theta_{2}} \\ \frac{\partial f}{\partial\theta_{1}}\frac{\partial f}{\partial\theta_{2}} & \left( \frac{\partial f}{\partial\theta_{2}} \right)^{2} \end{matrix} \right]\left\{ \begin{matrix} \dot{\theta}_{1} \\ \dot{\theta}_{2} \end{matrix} \right\} \right)$$

Equation 1

In the next page we will use the kinematics of the double inverted pendulum to calculate $f(\theta_{1}, \theta_{2})$. Doing so allows us to use Equation 10 to calculate the angular acceleration of the stance ankle-COM line.

| 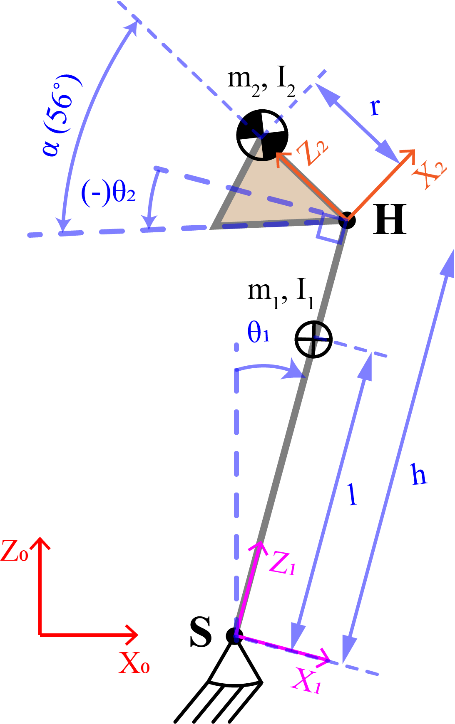 |
| --- |

$$\left( m_{1}+m_{2} \right) \vec{r}_{COM}=m_{1}\vec{r}_{m_{1}}+m_{2}\vec{r}_{m_{2}}$$

$$\left( m_{1}+m_{2} \right) \vec{r}_{COM}=m_{1}l \hat{k}_{1}+m_{2}(h \hat{k}_{1}+r \hat{k}_{2})$$

$$\left( m_{1}+m_{2} \right) \vec{r}_{COM}=\left( m_{1}l+m_{2}h \right) \hat{k}_{1}+m_{2}r \hat{k}_{2}$$

$$\left( m_{1}+m_{2} \right) \vec{r}_{COM}=\left( m_{1}l+m_{2}h \right) \left( \sin\left( \theta_{1} \right)\hat{i}_{0}+\cos\left( \theta_{1} \right)\hat{k}_{0} \right)+m_{2}r\left( -\cos\left( \theta_{1}+\theta_{2}+\alpha\right)\hat{i}_{0}+\sin\left( \theta_{1}+\theta_{2}+\alpha\right)\hat{k}_{0} \right)$$

$$f\left( \theta_{1}, \theta_{2} \right)=\tan\left( \theta\right)=\frac{r_{x}}{r_{z}}$$

$$f\left( \theta_{1}, \theta_{2} \right)=\tan\left( \theta\right)= \frac{\left( m_{1}l+m_{2}h \right)\sin\left( \theta_{1} \right)-m_{2}r\cos\left( \theta_{1}+\theta_{2}+\alpha\right)}{\left( m_{1}l+m_{2}h \right)\cos\left( \theta_{1} \right)+m_{2}r\sin\left( \theta_{1}+\theta_{2}+\alpha\right)}$$

Replace $\boldsymbol{P=}\left( \boldsymbol{m}_{\boldsymbol{1}}\boldsymbol{l+}\boldsymbol{m}_{\boldsymbol{2}}\boldsymbol{h} \right),$ $\boldsymbol{Q=}\boldsymbol{m}_{\boldsymbol{2}}\boldsymbol{r}$ then

$$f\left( \theta_{1}, \theta_{2} \right)=\tan\left( \theta\right)= \frac{P\sin\left( \theta_{1} \right)-Q\cos\left( \theta_{1}+\theta_{2}+\alpha\right)}{P\cos\left( \theta_{1} \right)+Q\sin\left( \theta_{1}+\theta_{2}+\alpha\right)}$$

$$1+f^{2}=\frac{P^{2}+Q^{2}+2PQ\sin(\theta_{2}+\alpha)}{\left( P\cos\left( \theta_{1} \right)+Q\sin\left( \theta_{1}+\theta_{2}+\alpha\right) \right)^{2}}$$

Calculate the partial derivatives of $f\left( \theta_{1}, \theta_{2} \right)$:

$$\frac{\partial f}{\partial\theta_{1}}= \frac{\left[ P\cos\left( \theta_{1} \right)+Q\sin\left( \theta_{1}+\theta_{2}+\alpha\right) \right]^{2}+{[P\sin\left( \theta_{1} \right)-Q\cos\left( \theta_{1}+\theta_{2}+\alpha\right)]}^{2}}{{[P\cos\left( \theta_{1} \right)+Q\sin\left( \theta_{1}+\theta_{2}+\alpha\right)]}^{2}}=1+f^{2}$$

$$\frac{\partial^{2}f}{\partial{\theta_{1}}^{2}}=2f\frac{\partial f}{\partial\theta_{1}}=2f (1+f^{2})$$

$$\frac{\partial f}{\partial\theta_{2}}=\frac{Q\sin\left( \theta_{1}+\theta_{2}+\alpha\right)\times\left[ P\cos\left( \theta_{1} \right)+Q\sin\left( \theta_{1}+\theta_{2}+\alpha\right) \right]-Q\cos\left( \theta_{1}+\theta_{2}+\alpha\right)\times\left[ P\sin\left( \theta_{1} \right)-Q\cos\left( \theta_{1}+\theta_{2}+\alpha\right) \right]}{{[P\cos\left( \theta_{1} \right)+Q\sin\left( \theta_{1}+\theta_{2}+\alpha\right)]}^{2}}$$

$$\frac{\partial f}{\partial\theta_{2}}=\frac{Q^{2}+PQ\sin(\theta_{2}+\alpha)}{{[P\cos\left( \theta_{1} \right)+Q\sin\left( \theta_{1}+\theta_{2}+\alpha\right)]}^{2}}$$

$$\frac{\partial^{2}f}{\partial\theta_{1}\partial\theta_{2}}=2f\frac{\partial f}{\partial\theta_{2}}$$

$$\frac{\partial^{2}f}{\partial{\theta_{2}}^{2}}=\frac{PQ\cos(\theta_{2}+\alpha)\left[ P\cos\left( \theta_{1} \right)+Q\sin\left( \theta_{1}+\theta_{2}+\alpha\right) \right]^{2}-2 \left[ P\cos\left( \theta_{1} \right)+Q\sin\left( \theta_{1}+\theta_{2}+\alpha\right) \right]Q\cos\left( \theta_{1}+\theta_{2}+\alpha\right)[Q^{2}+PQ\sin(\theta_{2}+\alpha)]}{{[P\cos\left( \theta_{1} \right)+Q\sin\left( \theta_{1}+\theta_{2}+\alpha\right)]}^{4}}$$

$$\frac{\partial^{2}f}{\partial{\theta_{2}}^{2}}=\frac{PQ\cos(\theta_{2}+\alpha)}{{[P\cos\left( \theta_{1} \right)+Q\sin\left( \theta_{1}+\theta_{2}+\alpha\right)]}^{2}}-\frac{2 Q\cos\left( \theta_{1}+\theta_{2}+\alpha\right)}{\left[ P\cos\left( \theta_{1} \right)+Q\sin\left( \theta_{1}+\theta_{2}+\alpha\right) \right]}.\frac{\partial f}{\partial\theta_{2}}$$

If we are in a quasistatic OLB state, we can move the COM medially or laterally using a moment at the ankle or hip in the frontal plane. To decide which direction to exert the control we have to combine Double Inverted Pendulum Equation 3 and Equation 10. First Equation 1:

$\ddot{\theta}=\frac{1}{\left( 1+f^{2} \right)}\left[ \begin{matrix} \frac{\partial f}{\partial\theta_{1}} & \frac{\partial f}{\partial\theta_{2}} \end{matrix} \right]\left\{ \begin{matrix} \ddot{\theta}_{1} \\ \ddot{\theta}_{2} \end{matrix} \right\}+\frac{1}{\left( 1+f^{2} \right)}\left( \left\{ \begin{matrix} \dot{\theta}_{1} & \dot{\theta}_{2} \end{matrix} \right\}\left[ \begin{matrix} \frac{\partial^{2}f}{{\partial\theta_{1}}^{2}} & \frac{\partial^{2}f}{\partial\theta_{1}\partial\theta_{2}} \\ \frac{\partial^{2}f}{\partial\theta_{1}\partial\theta_{2}} & \frac{\partial^{2}f}{{\partial\theta_{2}}^{2}} \end{matrix} \right]\left\{ \begin{matrix} \dot{\theta}_{1} \\ \dot{\theta}_{2} \end{matrix} \right\} \right)-\frac{2f}{\left( 1+f^{2} \right)^{3}}\left( \left\{ \begin{matrix} \dot{\theta}_{1} & \dot{\theta}_{2} \end{matrix} \right\}\left[ \begin{matrix} \left( \frac{\partial f}{\partial\theta_{1}} \right)^{2} & \frac{\partial f}{\partial\theta_{1}}\frac{\partial f}{\partial\theta_{2}} \\ \frac{\partial f}{\partial\theta_{1}}\frac{\partial f}{\partial\theta_{2}} & \left( \frac{\partial f}{\partial\theta_{2}} \right)^{2} \end{matrix} \right]\left\{ \begin{matrix} \dot{\theta}_{1} \\ \dot{\theta}_{2} \end{matrix} \right\} \right)\to$,

$\ddot{\theta}=\frac{1}{\left( 1+f^{2} \right)}\left[ \begin{matrix} \frac{\partial f}{\partial\theta_{1}} & \frac{\partial f}{\partial\theta_{2}} \end{matrix} \right]\left\{ \begin{matrix} \ddot{\theta}_{1} \\ \ddot{\theta}_{2} \end{matrix} \right\}+g\left( \theta_{1}, \theta_{2},\dot{\theta}_{1},\dot{\theta}_{2},P,Q \right)$

Now Double Inverted Pendulum Equation 3:

$$\left\{ \begin{matrix} \ddot{\theta}_{1} \\ \ddot{\theta}_{2} \end{matrix} \right\}=\left[ MM \right]^{-1}\left[ BB \right]\left\{ \begin{matrix} T_{1} \\ T_{2} \end{matrix} \right\}+\left[ MM \right]^{-1}\left\{ CC \right\}$$

Then when combined we have:

$$\ddot{\theta}=\frac{1}{\left( 1+f^{2} \right)}\left[ \begin{matrix} \frac{\partial f}{\partial\theta_{1}} & \frac{\partial f}{\partial\theta_{2}} \end{matrix} \right]\left[ MM \right]^{-1}\left[ BB \right]\left\{ \begin{matrix} T_{1} \\ T_{2} \end{matrix} \right\}+\frac{1}{\left( 1+f^{2} \right)}\left[ \begin{matrix} \frac{\partial f}{\partial\theta_{1}} & \frac{\partial f}{\partial\theta_{2}} \end{matrix} \right]\left[ MM \right]^{-1}\left\{ CC \right\}+g(\theta_{1}, \theta_{2},\dot{\theta}_{1},\dot{\theta}_{2},P,Q)$$

Equation 2- Angular acceleration of the stance foot-COM line for a double inverted pendulum

$\left[ MM \right]=\left[ \begin{matrix} I_{1}+m_{1}l^{2}+m_{2}h^{2}+m_{2}rh\sin\left( \theta_{2}+\alpha\right) & m_{2}rh\sin\left( \theta_{2}+\alpha\right) \\ I_{2}+m_{2}r^{2}+m_{2}hr\sin\left( \theta_{2}+\alpha\right) & \left( I_{2}+m_{2}r^{2} \right) \end{matrix} \right]$,

$\left[ BB \right]=\left[ \begin{matrix} 1 & -1 \\ 0 & 1 \end{matrix} \right]$,

$\left\{ CC \right\}=\left\{ \begin{matrix} \left( m_{1}l+m_{2}h \right)g\sin\left( \theta_{1} \right)-m_{2}rh\cos\left( \theta_{2}+\alpha\right)\left( \dot{\theta}_{1}+\dot{\theta}_{2} \right)^{2} \\ -m_{2}gr\cos\left( \theta_{1}+\theta_{2}+\alpha\right)+m_{2}hr\cos\left( \theta_{2}+\alpha\right){\dot{\theta}_{1}}^{2} \end{matrix} \right\}$,

***4. Strategies for creating maximum COM acceleration in the medial and lateral directions during OLB:***

Regardless of our initial state, one’s ability to affect control over the dynamics of the OLB is limited to the first term in Equation 2. The other terms represent inertial forces due to gravity and current momentum of the body.

$\left[ GG \right]\left[ MM \right]^{-1}\left[ BB \right]\left\{ \begin{matrix} T_{1} \\ T_{2} \end{matrix} \right\}=\left[ \begin{matrix} K_{Ankle} & K_{Hip} \end{matrix} \right]\left\{ \begin{matrix} T_{1} \\ T_{2} \end{matrix} \right\}$, where

*P = m_1_ l + m_2_ h* and *Q = m_2_ r*

$$f\left( \theta_{1}, \theta_{2} \right)=\tan\left( \theta\right)= \frac{P\sin\left( \theta_{1} \right)-Q\cos\left( \theta_{1}+\theta_{2}+\alpha\right)}{P\cos\left( \theta_{1} \right)+Q\sin\left( \theta_{1}+\theta_{2}+\alpha\right)}$$

$$\left[ GG \right]=\frac{1}{\left( 1+f^{2} \right)}\left[ \begin{matrix} \frac{\partial f}{\partial\theta_{1}} & \frac{\partial f}{\partial\theta_{2}} \end{matrix} \right]=\frac{1}{\left( 1+f^{2} \right)}\left[ \begin{matrix} \left( 1+f^{2} \right) & \left( \frac{Q^{2}+PQ\sin\left( \theta_{2}+\alpha\right)}{\left[ P\cos\left( \theta_{1} \right)+Q\sin\left( \theta_{1}+\theta_{2}+\alpha\right) \right]^{2}} \right) \end{matrix} \right]\to$$

$$\left[ GG \right]=\left[ \begin{matrix} 1 & \left( \frac{Q^{2}+PQ\sin\left( \theta_{2}+\alpha\right)}{P^{2}+Q^{2}+2PQ\sin\left( \theta_{2}+\alpha\right)} \right) \end{matrix} \right]$$

$\left[ MM \right]=\left[ \begin{matrix} I_{1}+m_{1}l^{2}+m_{2}h^{2}+m_{2}rh\sin\left( \theta_{2}+\alpha\right) & m_{2}rh\sin\left( \theta_{2}+\alpha\right) \\ I_{2}+m_{2}r^{2}+m_{2}hr\sin\left( \theta_{2}+\alpha\right) & I_{2}+m_{2}r^{2} \end{matrix} \right]$,

$$\left[ BB \right]=\left[ \begin{matrix} 1 & -1 \\ 0 & 1 \end{matrix} \right]$$

We calculated $\left[ \begin{matrix} K_{Ankle} & K_{Hip} \end{matrix} \right]$ for all the states within the ankle and hip ranges of motion (-17º < θ_1_ < 11º and -31º < θ_2_ < 53 º). The signs of K_Ankle_ and K_Hip_ do not change. If we partition the range of motion to 0.1 degree increments and calculate K_Ankle_ and K_Hip_ we get the following summary:

$$\left[ \begin{matrix} K_{Ankle}\pm\left( SD \right) & K_{Hip}\pm\left( SD \right) \end{matrix} \right]=[\begin{matrix} 0.015\pm(2e-4) & -0.0147\pm(6e-4) \end{matrix}]$$

So at any state of OLB, to move the COM laterally ($\ddot{\theta}>0$), we should increase ankle moment (take the COP towards the medial margin of BOS) and decrease hip abduction moment (decrease the pelvic inclination angle). Reversely, if we want to move the COM medially ($\ddot{\theta}<0$) *e.g.* to avoid a lateral fall on the hip, we should decrease the ankle moment (take the COP toward the lateral margin of BOS) and increase hip abduction moment (increase the pelvic inclination angle).
