# Supplementary material for: On the importance of the hip abductors during a clinical one legged balance test: A theoretical study
Source: PLoS One. 2020 Nov 13;15(11):e0242454. doi: 10.1371/journal.pone.0242454 (PMC7665826; doi:10.1371/journal.pone.0242454)
Supplement: S3 Text — (DOCX) [file pone.0242454.s003.docx]

**S3 Text: Simulation of the planar movements of a double inverted pendulum model with range of motion constraints**

Equations of motion for the double inverted pendulum model were derived in S2 Text. To simulate the movements of the model, we can set up our equations in the following manner:

$X={(\theta_{1},\dot{\theta}_{1},\theta_{2},\dot{\theta}_{2})}^{'}\to\dot{X}={(\dot{\theta}_{1},\ddot{\theta}_{1},\dot{\theta}_{2},\ddot{\theta}_{2})}^{'}$,

where we replace the values for ($\ddot{\theta}_{1},\ddot{\theta}_{2}$) from the derived equations of motion in S2 Text. Numerically solving the above differential equation, using the *ode45* function in MATLAB © is common in many fields. So we will not include it here. However, there are certain assumptions that need clarification. In the following sections, the important assumptions that were used in solving the above differential equation in MALAB are discussed.

***1. Enforcing the end of ROM constraint for the stance hip***

Enforcing the end of range of motion (ROM) for the stance hip is very important for the results in Figure 5 of the main paper. The large hip abduction / adduction moments can rapidly change the angular momentum of the upper body and therefore induce a large hip angular acceleration. However, this also means that using the hip strategy will cause the person to reach the end of ROM of the stance hip quickly. When the joint angle gets closer to the end of ROM, the ligaments surrounding the hip will engage to exert a counter moment.

In our simulations, we divided the hip moment to two parts: T_Active_ and T_Passive_. The first part is the muscle-equivalent hip abduction / adduction moment. Since in our simulations, we were using the maximum COM acceleration strategy, the T_Active_ value was set to reach the maximum hip abduction moment from its initial value, based on the enforced rate of torque development (See Methods in the main paper). We also included the force-length characteristics of the muscular isometric hip abduction as discussed in the Methods. On the other hand the second part, T_Passive_, was further divided to two components that were added together: first, the exponential elastic component presented in the paper (T_Passive1_), and second, a damping term applied within the last two degrees of the ROM ($T_{Passive2}=-b(\theta_{2})\times\dot{\theta}_{2}$). T_Passive2_ essentially stops the hip angle from passing the end of ROM. We set b to be close to zero in the middle of the ROM and increase exponentially near the two ends of ROM to a large constant value. The function for b can be seen in Figure 1.

| 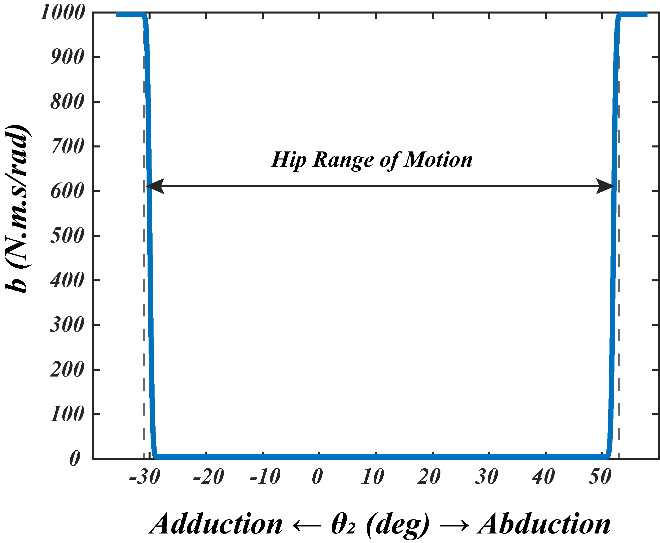  **Figure 1-** Coefficient of Damping (b(θ_2_)) considered for calculating $T_{Passive2}=-b(\theta_{2})\times\dot{\theta}_{2}$, which enforced the end of hip ROM. Our choice of 1000 for the maximum b and 2 degrees for the rise of b value from zero was arbitrary. However, we checked for the sensitivity of the recoverable OLB states to these choices (1000 vs 500, and 2 degrees vs 5 degrees) and did not observe a noticeable change in results. |
| --- |

***2. Enforcing the ROM constraint for the stance ankle***

If we consider the foot to be glued to the ground, then θ_1_ is the ankle eversion angle. We used the end of ankle ROM as the termination criterion for the integration algorithm. This was based on the simple notion that given the narrow range of θ_1_ values seen in the FBR, should θ_1_ reach such high deviations, the balance should already be passed the point of recovery by either the hip or ankle strategies.

***3. Criterion for considering an initial quasistatic OLB state as recoverable***

Starting from each quasistatic state within the hip and ankle ROM, we ran two simulations: once with maximum ankle inversion and maximum hip abduction moments to induce the maximum medial acceleration of the COM; then, we reversed the ankle and hip moment signs to create the maximum lateral acceleration of COM. In terms of our criterion for recoverability of an initial OLB state, we posit that if one could induce a fall to both lateral and medial sides from an initial state, then there should be a control strategy which could bring the COM over the ankle. In terms of our simulations results, this translates to the sign of θ upon termination of the simulation (the angle of the line connecting the stance ankle to the COM of the whole body) being different between the two different simulation runs.
