# Supplementary material for: On the importance of the hip abductors during a clinical one legged balance test: A theoretical study
Source: PLoS One. 2020 Nov 13;15(11):e0242454. doi: 10.1371/journal.pone.0242454 (PMC7665826; doi:10.1371/journal.pone.0242454)
Supplement: S4 Text — (DOCX) [file pone.0242454.s004.docx]

**S4 Text: Predicted sensitivity of active FBR and hip abduction moment to medio-lateral bending of the trunk and movements of the contralateral leg in the frontal plane during OLB**

In our calculations we assumed the subjects would keep their trunk straight and their contralateral leg at a zero hip abduction angle during one-legged balance (OLB). Given these assumptions, we then proceeded to calculate *m_2_*, *α*, and *r* (please see Figure 1 in the manuscript). These parameters describe the anthropometry and mass distribution of the top link in the double inverted pendulum model. The mass of the top link (*m_2_*) does not change. However, *α* and *r* do vary with changes in the contralateral hip abduction angle and medio-lateral bending angle of the trunk. In what follows, we will present our method for calculating the sensitivity of the estimated hip abduction moment demand of OLB and the active FBR to the induced changes in *α* and *r*.

| 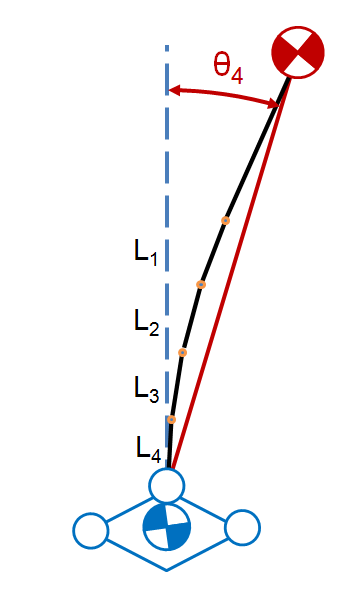  **Figure 1-** Maximum lateral bending of the center of mass of the head, arms, and trunk due to the lateral bending in lumbar vertebrae (θ_4_). Thorax was considered rigid because of the added stiffness due to the rib cage. |
| --- |

***Calculating the sensitivity of the results to changes to the contralateral hip abduction angle during OLB***

Given the large mass of the contralateral leg, the subject can increase their contralateral hip abduction angle to affect their OLB and therefore FBR. To calculate the sensitivity of the FBR to this degree of freedom, we allowed a -10 to 30 degrees hip abduction angle at the contralateral leg in our sensitivity calculations for the FBR.

***Calculating the sensitivity of active FBR to lateral bending of the trunk during OLB***

| 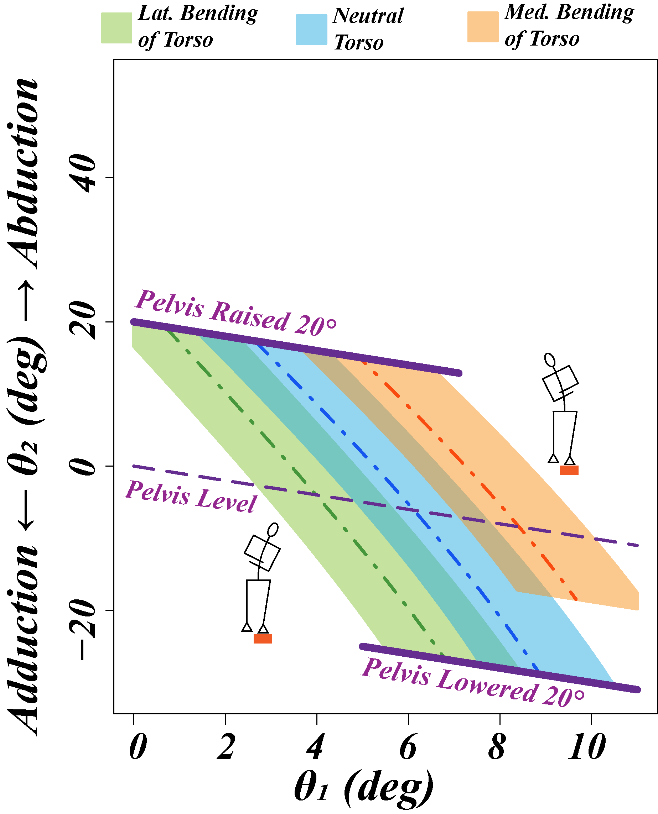  **Figure 2-** Predicted effect of ±10° medio-lateral bending of the lumbar spine on the active FBR of the 50^th^ percentile healthy old man. We can see that a medial (lateral) bending of the lumbar spine shifts the FBR to the right (left). The width of the FBR stays essentially unchanged. Medial bending of the lumbar spine increases the required hip abduction moment for OLB. If the hip abductor muscles are not strong enough, this results in shortening of the FBR. Conversely, lateral bending of the lumbar spine decreases the hip abduction moment and could increase the length of the FBR in the case of weak abductor muscles. |
| --- |

Given the high mass of the head, arms, and trunk we can expect the FBR to be highly sensitive to any lateral bending of spine. We used published data on the maximal lateral bending of the vertebrae in the lumbar spine (1) to estimate the maximum lateral bending that the mass of head, arms, and trunk could experience relative to the pelvis (θ_4_ ~ 20 degrees in Figure 1). In our calculations for the sensitivity of the FBR to lateral bending of the trunk, we used half of this value as a reasonable range to expect during normal OLB.

**Results:**

Table 1 shows the effect of ±10° of medio-lateral bending of the torso and a variation from -10° to 30° of contralateral (Cont.) hip abduction on the parameters determining the effective moment arm of m_2_ for the model of the 50^th^ percentile man. We can see that laterally bending the torso and adducting the contralateral leg decrease the effective moment arm, while medially bending the torso and abducting the contralateral leg increase it.

Figure 2 shows the effect of ±10 degree of medio-lateral bending of the lumbar spine on the FBR for healthy old men. The width of FBR is unaffected because it is determined by the width of the functional BOS. On the other hand, the increase in the demand for hip abduction moment due to medially bending the torso shortens the FBR. If the hip abduction strength was large enough, the medial (lateral) bending of the spine would just shift the FBR to the right (left) between the two parallel lines set by ±20° pelvic inclination angle increase and decrease.

| **Table 1-** Predicted sensitivity of the calculated α, r, and hip abduction moment to ±10 degree changes in medio-lateral bending of the lumbar spine and -10° and 30° of contralateral hip abduction for the mid-size man double inverted pendulum model. For the definition of r and α, please refer to Fig. 1C in the manuscript.   \|  \| ***Case 1*** \| ***Case 2*** \| ***Case 3*** \| ***Case 4*** \| ***Case 5*** \| \| --- \| --- \| --- \| --- \| --- \| --- \| \| ***Lumbar Spine Medio-Lateral Bending Angle (deg)*** \| ***0*** \| ***10*° *Lateral*** \| ***10*° *Medial*** \| ***0*** \| ***0*** \| \| ***Contralateral Hip Abduction Angle (deg)*** \| ***0*** \| ***0*** \| ***0*** \| ***30*** \| ***-10*** \| \| ***r (cm)*** \| 19 \| 17 \| 21 \| 22 \| 18 \| \| ***α (deg)*** \| 56 \| 66 \| 46 \| 49 \| 59 \| \| ***OLB Hip Abduction Moment Demand (N.m)*** \| 70 \| 44 \| 96 \| 95 \| 61 \| \| ***% Increase in OLB Hip Abd Moment*** \| - \| -37 \| 37 \| 36 \| -13 \| |
| --- | --- | --- | --- | --- | --- | --- | --- | --- | --- | --- | --- | --- | --- | --- | --- | --- | --- | --- | --- | --- | --- | --- | --- | --- | --- | --- | --- | --- | --- | --- | --- | --- | --- | --- | --- | --- | --- | --- | --- | --- | --- | --- |

**References:**

1. Schultz AB, Ashton-Miller JA. “Biomechanics of the Human Spine” in Basic Orthopaedic Biomechanics [Internet]. 1st ed. Mow VC, Hayes WC, editors. New York: Raven Press; 1991. 351 p. Available from: https://books.google.com/books?id=vkxRAAAAMAAJ
